# Brain structural and functional connectivity converge prenatally but diverge after birth

**DOI:** 10.1371/journal.pbio.3003927

**Published:** 2026-09-09

**Authors:** Ruoke Zhao, Mingyang Li, Yuqi Zhang, Ruike Chen, Chenglin Ning, Zhiyong Zhao, Zhihan Yan, Meihao Wang, Dan Wu

**Affiliations:** 1 College of Biomedical Engineering & Instrument Science, Zhejiang University, Hangzhou, China; 2 Children’s Hospital, Zhejiang University School of Medicine, National Clinical Research Center for Children and Adolescents’ Health and Diseases, Hangzhou, China; 3 Department of Radiology, The Second Affiliated Hospital and Yuying Children’s Hospital of Wenzhou Medical University, Wenzhou, China; 4 Key Laboratory of Intelligent Medical Imaging of Wenzhou, The First Affiliated Hospital of Wenzhou Medical University, Wenzhou, China; Inserm U1208, FRANCE

## Abstract

Brain anatomical architecture supports its functional activity and complex cognitive processes. However, how the structure–function (SF) relationship establishes and develops during early life, as well as its underlying mechanisms, remain largely unclear. To address these questions, we leveraged multimodal MRI data from two large-scale public databases, the developing Human Connectome Project (dHCP) and the Baby Connectome Project (BCP), to characterize the spatiotemporal dynamics of SF coupling from the perinatal period to toddlerhood. Our results revealed that SF coupling at birth exhibited a spatial variation along the sensorimotor-association cortical axis. During the perinatal period (26–44 postmenstrual weeks), SF coupling strengthened drastically and followed three distinct developmental trajectories across the cortex, with sensorimotor and visual areas showing the fastest growth and the earliest plateau. After birth, SF coupling shifted toward a weakening pattern across the cortex during infancy and toddlerhood (1–28 months). These developmental changes of SF coupling were more strongly associated with the maturation of functional connectivity, which first converged toward the local structural architecture prenatally and then diverged postnatally through the expansion of global inter-modular pathways. Furthermore, SF coupling at birth, the developmental transition point, exhibited a significant association with individual differences in cognition and language outcomes at 18 months of age. Collectively, these findings offer valuable insights into the organizational principles underlying structural and functional network development during early life as well as the complex evolving relationship between them.

## Abstract 2

How do the brain’s anatomical architecture and functional activity patterns become established during early life? This study characterizes the brain’s developmental changes from the perinatal period to toddlerhood, offering insights into the organization of structural and functional networks and cognitive development.

## Introduction

The human brain undergoes rapid and intricate changes during early life, particularly from the prenatal period through the first few years of life. Within this window, a series of molecular and cellular processes unfold in succession, including neuronal proliferation and migration, and axonal growth primarily occurring in the fetal stage, followed by prolonged dendritic arborization, synaptogenesis, and axonal pruning and myelination [1–3]. Driven by these neurogenesis events, white matter pathways establish rapidly as the anatomical backbone [4,5] and large-scale functional organization progressively emerges [6,7]. Characterizing the complex developmental trajectories of brain structure and function, and their interrelationship during early life, is fundamental for uncovering the organizational principles underlying typical brain maturation.

Network neuroscience [8] using magnetic resonance imaging (MRI) offers a powerful framework for characterizing brain structural and functional organization. Previous studies have suggested that both structural and functional networks follow a similar developmental order, i.e., the primary sensorimotor cortices develop earlier than the higher-order association cortices [9]. On the other hand, there are considerably different developmental trajectories between the two networks. Structural network, typically derived through diffusion MRI-based tractography to quantify physical connections between brain regions, shows significantly enhanced short- and long-range connections during the prenatal period [10] and, by the time of birth, already exhibits an adult-like topology with rich–club organization [11,12]. Following birth, structural network undergoes further refinement, with the most prominent changes occurring in the connections of association regions [13,14]. In contrast, functional network, which captures inter-regional co-activation by calculating correlations of BOLD functional MRI signals between regions, follow a more gradual and prolonged course of development. Primary functional networks (e.g., visual, motor, and auditory) have been shown to be present in the brains of fetuses, preterm- and term-born infants, whereas higher-order networks (e.g., default mode network, DMN) remain immature or incomplete [6,7,15–17] and continue to strengthen throughout childhood and adolescence [18,19]. This consistent sequence yet asynchronous timeline between structural and functional development prompts further investigate into their relationship. Does the functional connectome closely follow the developmental trajectory of the structural connectome? Does their relationship become more tightly tethered with development or the other way around? Direct evidence elucidating this relationship remains notably limited during early life.

In recent years, structure–function (SF) coupling has been proposed as a biomarker to quantify the extent to which a region’s functional activity statistically depends to its underlying structural wiring [20]. It is typically defined as the Spearman’s correlation coefficient between a brain region’s structural connectivity vector and its functional connectivity vector [21–25]. Previous studies have examined the spatial heterogeneity of SF coupling across the adult cortex with several established hierarchies, such as the primary functional gradient [26] and cyto-architecture organization [27]. Specifically, structural and functional connectivities are more strongly coupled in unimodal and agranular cortices than the transmodal and granular regions [23,28,29]. Beyond regional heterogeneity, SF coupling has been shown to effectively capture individual differences in cognitive abilities and task performance [21,22,30–32], as well as pathological brain alterations across a wide range of neurological and psychiatric disorders [33–39].

From a developmental perspective, SF coupling exhibits region-specific and nonlinear changes across the entire course of brain maturation. Several studies spanning school age to early adulthood (ages 5–23 years) have highlighted a heterogeneous pattern of SF coupling development across the cortex. For instance, the higher-order functional networks (e.g., frontoparietal network [FPN] and default mode network [DMN]) showed marked increases, while primary sensory networks remain relatively stable or even exhibit slight decreases from late childhood to early adulthood [20,22,40]. In early childhood, a longitudinal study that collected data at ages 1, 2, 4, and 6 reported an overall declining trend in SF coupling, with the most widespread reductions observed between 1 and 2 years [41]. This decline in SF coupling during childhood was further supported by another study with data sampled at 4.5, 6, and 7.5 years of age [25]. In the neonatal period, a recent study involving healthy term-born neonates found that SF coupling in primary sensory systems was negatively associated with age during the first month after birth [42]. As for the perinatal period, two studies comparing the coupling between preterm infants at birth and at term-equivalent age (TEA) have consistently reported widespread cortical increases in SF coupling [43,44]. Despite these existing insights, the detailed spatiotemporal dynamics of SF coupling across perinatal and early postnatal development in large cohorts remain largely unknown.

To address this gap, we investigated SF coupling in two large public datasets, the Developing Human Connectome Project (dHCP) and the Baby Connectome Project (BCP), covering the perinatal period, infancy and toddlerhood, using an infant-specific cortical atlas. We first examined the spatial pattern of SF coupling at birth in comparison with that of adults and well-established cortical hierarchies. Next, we employed non-negative matrix factorization (NMF) to capture spatially heterogeneous development across the cortex, and then characterized SF coupling trajectories from 26 postmenstrual weeks to 28 postnatal months. To characterize the relative contributions of structural and functional networks to the alterations in SF coupling, we examined the development of both structural and functional network topologies and their associations with SF coupling. We hypothesized that, given that the structural network develops and stabilizes early, the dynamics of the functional network serve as the dominant contributor to the developmental shift in SF coupling. Finally, we assessed the longitudinal association between neonatal SF coupling and cognition outcomes at 18 months of age. Through these analyses, we sought to delineate the spatiotemporal trajectory of SF coupling during early brain development and to uncover the dynamic mechanisms that shape its development.

## Materials and methods

### Participants and inclusion criteria

**Fig 1:**
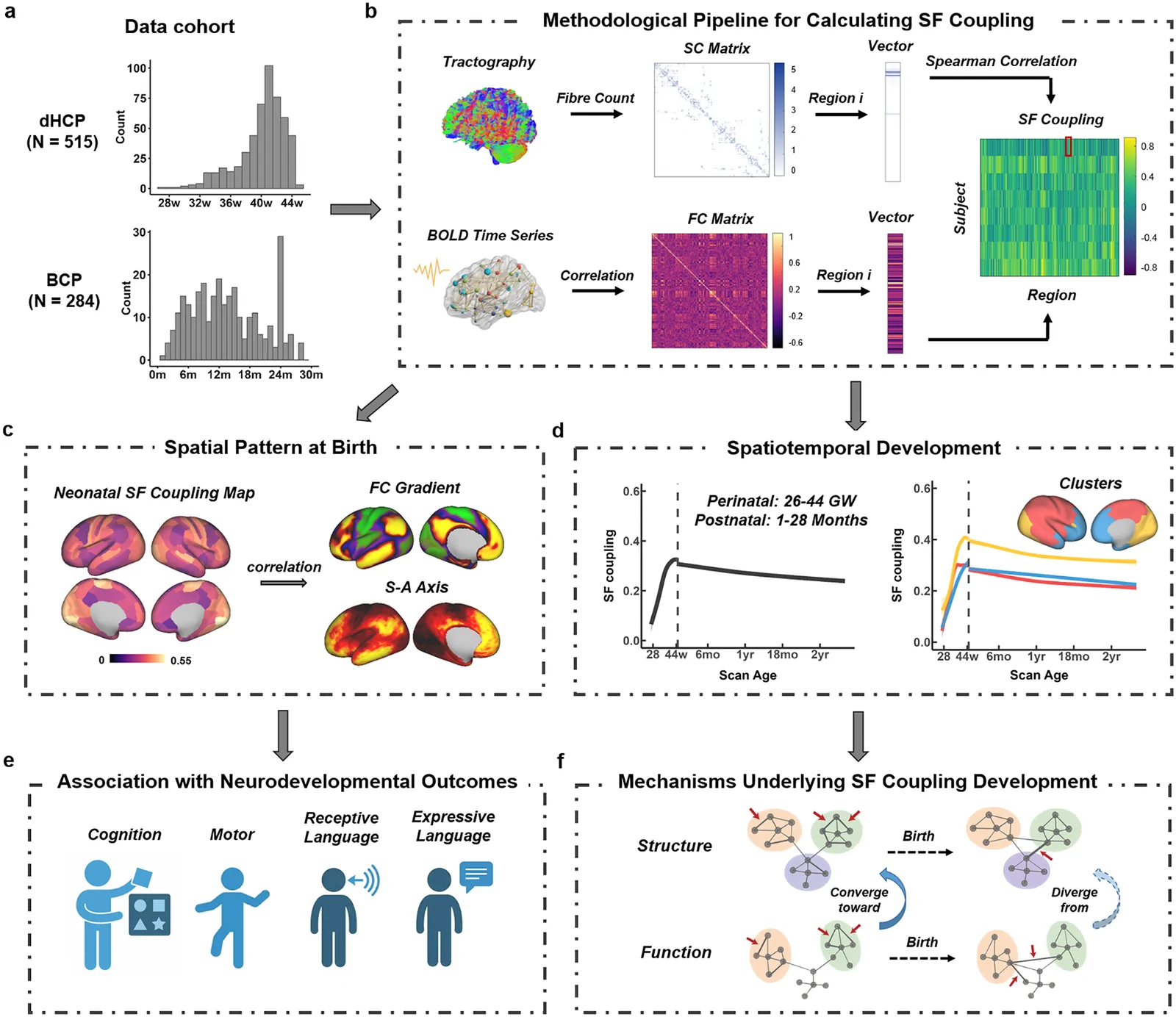
The workflow of this study. **(a)** Age distribution of subjects in the two cohorts. **(b)** Methodological pipeline for calculating SF coupling. We first examined the spatial pattern of SF coupling at birth **(c)**, then characterized spatiotemporal developmental trajectories of SF coupling from 26 postmenstrual weeks to 28 postnatal months **(d)**. We further explored the mechanisms underlying SF coupling development by characterizing the age-related changes of structural and functional network topologies **(e)**. Finally, we assessed the longitudinal association between neonatal SF coupling and cognition outcomes at 18 months of age **(f)**.

The dHCP release 3 dataset comprises 887 scans from 783 preterm- or term-born neonates, with gestational age (GA) at birth ranging from 23 to 43.57 weeks and scan age between 26.71 and 45.14 weeks (https://www.developingconnectome.org/project/). Subjects were excluded based on the following criteria (S1a Fig): (1) sedation during scanning; (2) radiology scores >2, as assessed by perinatal neuroradiologists, indicating visual brain abnormality radiologically; (3) missing any required MRI modality (T2-weighted, diffusion or resting-state functional MRI); (4) failure in any preprocessing pipeline; (5) failure in cortical surface registration; or (6) severe head motion during scanning (mean frame-wise displacement [FD] > 0.5). A total of 515 scans from 484 neonates (GA at birth: 23.71–42.29 weeks; scan age: 26.71–44.86 postmenstrual weeks) were finally included (see Figs 1a and S2a for age and longitudinal distributions).

For the BCP, typically developing infants, toddlers, and preschool-aged children were recruited and scanned between birth and 5 years of age (https://nda.nih.gov/edit_collection.html?id=2848) [45]. Participants were excluded if they (1) were missing any MRI modality (T1-weighted, diffusion or resting-state functional MRI); (2) showed structural abnormalities or poor image quality on visual inspection; (3) failed any preprocessing pipeline; (4) failed multimodal registration; or (5) had severe head motion (mean FD > 0.5); or (6) were older than 28 months at the time of scanning due to the limited sample size between 28 months and 5 years (*N* = 22) (S1b Fig). A total of 284 scans from 164 participants were retained (see Figs 1a and S2b for age and longitudinal distributions).

In addition, 98 healthy participants from the HCP-YA dataset [46] were included. All participants had complete preprocessed data across the three MRI modalities and showed no severe head motion.

### Image acquisition

MRI of the dHCP subjects was performed on a 3T Philips Achieva scanner using a dedicated neonatal system with 32-channel head coil at St. Thomas Hospital, London [47]. T2-weighted images were acquired with repetition time (TR) = 12,000 ms, echo time (TE) = 156 ms, SENSE factor of 2.11 (axial) and 2.60 (sagittal), and a resolution of 0.8 × 0.8 × 1.6 mm. Resting-state functional MRI (rs-fMRI) data were collected using multiband (MB) 9× accelerated echo-planar imaging (EPI) with TR/TE = 392/38 ms, 2,300 volumes and 2.15 mm isotropic resolution. Diffusion MRI (dMRI) acquisition parameters were as follows: TR/TE = 3,800/90 ms, *b*-values = 0/400/1,000/2,600 s/mm^2^, with 20/64/88/128 diffusion directions, multiband (MB) factor = 4, SENSE factor = 1.2, partial Fourier factor = 0.86, and a spatial resolution of 1.5 × 1.5 × 3 mm.

For the BCP dataset, MRI scans were acquired on 3T Siemens Prisma scanners using a Siemens 32-channel head coil at the University of Minnesota and the University of North Carolina at Chapel Hill [45]. T1-weighted images were acquired using a 3D magnetization-prepared rapid gradient echo (3D-MPRAGE) sequence with TR/TE/TI = 2,400/2.24/1,060 ms and 0.8 mm isotropic resolution. rs-fMRI data were collected using a single-shot EPI sequence with TR/TE = 800/37 ms, 420 volumes, MB factor = 8, and 2 mm isotropic resolution. dMRI data were obtained using either a 2-shell or 6-shell protocol. The 2-shell protocol used TR/TE = 3,222/89.2 ms, *b*-values = 0/1,500/3,000 s/mm^2^, 6/37/37 diffusion directions, MB factor = 4, and 1.5 mm isotropic resolution. The 6-shell protocol used TR/TE = 2640/88.6 ms, *b*-values = 0/500/1,000/1,500/2,000/2,500/3,000 s/mm^2^, with 7/9/12/17/24/34/48 diffusion directions, MB factor = 5, and 1.5 mm isotropic resolution. To assess the effect of acquisition protocol on the results, the dMRI protocol was included as a covariate in subsequent analyses. While SF coupling showed no significant differences between the two protocols (S3 Fig), SC network metrics did differ significantly (S4 Fig). These differences were then regressed out using the 6-shell protocol as the reference.

For the HCP dataset, acquisition details are available in previous publications [48–50]. Briefly, T1-weighted images were acquired using 3D MPRAGE sequence with voxel size = 0.7 mm isotropic, TR/TE = 2,400/2.14 ms. T2-weighted images were collected using SPACE sequence with the same voxel size as T1, and TR/TE = 3,200/565 ms. rs-fMRI was acquired using gradient-echo EPI sequence with 1,200 volumes/run, voxel size = 2 mm isotropic, and TR/TE = 720/33.1 ms, and high angular resolution diffusion imaging was collected with voxel size = 1.25 mm isotropic, TR/TE = 5520/89.5 ms, *b*-values = 1,000/2,000/3,000 s/mm^2^, 90 directions per *b*-value, and 18 *b*_0_ acquisitions.

### Image preprocessing

dHCP multimodal images underwent minimal preprocessing, as detailed in previous papers [51–53]. In this study, minimally preprocessed T2-weighted images were directly employed for analysis. Additional processing was applied to rs-fMRI data using custom codes and the DPABI toolbox [54]: (1) selection of a continuous subset of 1,600 volumes (approximately 70% of the total time series) with the lowest mean FD; (2) rigid registration to T2-weighted space; (3) linear detrending; and (4) regression of nuisance covariates, including head motion parameters, and white matter, CSF, and whole brain signals. For the dMRI data, the 4D volumes were first registered to T2-weighted space. Fiber orientation distributions (FODs) were estimated using single tissue constrained spherical deconvolution (ST-CSD) [55] in MRtrix3 (https://www.mrtrix.org/). Whole-brain probabilistic tractography was then performed using the iFOD2 [56] algorithm and the Anatomically-Constrained Tractography (ACT) [57] framework with the following parameters: step size = 0.5, length = 10–250 mm, cutoff = 0.06, maximum angle = 22.5 and 5,000k streamlines. The resulting streamlines were filtered to 1,000k using SIFT [58]. The minimally preprocessed HCP data also underwent the same additional processing as in the dHCP.

For the BCP dataset, structural MRI images were preprocessed using the Infant Brain Extraction and Analysis Toolkit (iBEAT V2.0 Cloud) [59], including N3 field inhomogeneity correction, skull stripping, T1–T2 alignment, and tissue segmentation. rs-fMRI data underwent the recommended pipeline for BCP [60], consistent with the HCP-style protocol [61], involving motion correction, EPI distortion correction, registration to T1, high-pass filtering, ICA-based denoising, and nuisance regression including head motion parameters as well as the signals from white matter, CSF, and the global brain. dMRI data was preprocessed using an infant-appropriate pipeline [62], including denoising, Gibbs ringing removal, EPI distortion correction, eddy current and slice-to-volume correction, bias field correction, and registration to T1. FODs were estimated using the multi-shell multi-tissue constrained spherical deconvolution (MSMT-CSD) algorithm [63] to leverage the well-distributed b-shells (up to *b* = 3,000) and high angular resolution of the BCP data. Whole-brain probabilistic tractography was subsequently performed with the same parameters as for the dHCP data. To evaluate the potential impact of using different FOD estimation methods across datasets, we performed a sensitivity analysis on a subsample (*N* = 50) from the BCP cohort using the ST-CSD approach. The results demonstrated that coupling values derived from ST-CSD and MSMT-CSD were highly correlated and revealed consistent developmental trajectories (see S5 Fig). Additional preprocessing details are provided in the Image Preprocessing of S1 Methods.

### Structure–function coupling

We chose our previously established neonatal atlas [64] (containing 210 regions) as the cortical parcellation template. This atlas integrates anatomical and functional signatures derived from multi-modal MRI data and has demonstrated stability and high interpretability. Detailed registration procedures from atlas space to individual space are described in the Cortical Parcellation Registration of S1 Methods [46,53,64–69].

The structural connection strength between each pair of regions was defined by the number of streamlines scaled by the inverse of the volumes of the two regions, yielding a symmetric structural connectivity matrix. The average raw densities of these matrices were as follows: 40.14% ± 3.15% for the dHCP dataset, 43.76% ± 2.06% for the HCP, and 29.06% ± 5.39% for the BCP. Here, density is defined as the proportion of existing edges (non-zero entries) relative to all possible edges in the matrix. Since network density can directly affect the calculation of topological metrics and SF coupling, we thresholded all matrices by enforcing a uniform density to standardize the network cost and ensure comparability across datasets. The primary analyses were conducted at a density of 25%, which was lower than the average raw density of each dataset and was selected to reduce the influence of weak and potentially spurious tractography-derived edges. Importantly, this threshold should be interpreted as an analytic choice rather than an estimate of the true biological density. To evaluate whether our findings were dependent on this density choice, additional analyses were performed using alternative density levels of 20% and 30%, as well as the unthresholded structural connectivity matrices (see Sensitivity analysis).

For the resting-state fMRI data, the BOLD signal was first spatially averaged across all voxels within each node to produce a representative time series for the entire scan. Subsequently, functional connection strength between each pair of regions was defined as the Pearson correlation coefficient between their respective time series, resulting in a fully connected symmetric matrix.

SF coupling was defined as the Spearman rank correlation coefficient between the nonzero elements of a region’s structural and functional connectivity profiles, consistent with previous studies [21,22,41]. To avoid bias, regions with fewer than 10 nonzero elements were excluded. Whole-brain SF coupling was calculated as the mean value across all brain regions.

### Network metrics

We next aimed to characterize the topological development of the structural and functional connectomes to provide a framework for understanding their contributions to SF coupling. Given that primary networks (e.g., visual and sensorimotor) are largely established by birth, as previously reported [6,7,11,12,15,16], it is of interest to examine how modular architectures reorganize toward higher-order maturation postnatally, and how this topological maturation influences SF coupling. To this end, we selected two network metrics, participation coefficient and modularity [70]. The participation coefficient quantifies the diversity of a given node’s connections across different modules [71], with a higher value indicating a stronger capacity for information integration. Module assignments were defined based on Yeo’s 7 network atlas. The whole-brain participation coefficient was calculated as the mean of all nodal participation coefficients. To assess segregation, we used modularity, which measures the extent to which a network can be partitioned into locally clustered modules [72]; higher modularity indicates a more pronounced modular organization. Specifically, for each network, modularity was calculated across 100 iterations using the Louvain algorithm. The final value was defined as the average modularity across iterations that yielded the most frequent (mode) number of communities. Network metrics for both structural and functional networks were computed using the Brain Connectivity Toolbox (https://github.com/brainlife/BCT). Detailed definitions, formulas, and computational procedures are provided in the Network Metrics of S1 Methods [71,72].

### Comparison SF coupling map with existing cortical hierarchies

To elucidate the spatial pattern of SF coupling at birth, we compared it with established cortical hierarchies, including the principal functional gradient [26], evolutionary surface area expansion [73], and the sensorimotor-association axis [74]. These hierarchies were projected from the HCP S1200 Group Average template surface (https://db.humanconnectome.org/data/projects/HCP_1200) [46] onto the dHCP 40-week template surface [65] using registration files from a prior study [66]. We calculated Pearson’s correlation coefficients between SF coupling and these cortical hierarchies across brain regions, with statistical significances determined through 1,000 spatial permutation tests [75]. Besides, each cortical region was assigned to one of the seven functional sub-network defined by Yeo and colleagues [76]. The mean SF coupling was then compared across networks.

### Non-negative matrix factorization

Non-negative matrix factorization was employed on regional SF coupling maps across subjects in the dHCP cohort to partitioning the brain into distinct clusters [77–79]. Since SF coupling ranged from −1–1, we first added 1 to all values to ensure non-negativity. A data matrix $V\in R^{m\times n}$, where m and n, respectively, denotes the number of subjects and regions, respectively, was used as the input for NMF and approximated as the product of two sparse matrices, W and H, by minimizing the following formulation


$$\begin{array}{c} \underset{W}{\text{min}}{\left\|V-WH\right\|}_{F}^{\text{2}} \end{array}$$

where W≥0 and H≥0. In the matrix $H\in R^{k\times n}$, each row represented loading coefficients of regions for one component, where k is the number of components. In the matrix $W\in R^{m\times k}$, each column represents subject-specific weights for one component. Subsequently, each region was assigned to one cluster corresponding to the highest loading coefficient, allowing for soft clustering. To determine the optimal number of components (clusters), we evaluated NMF performance for k values ranging from 2 to 10 using several metrics, including the Silhouette Coefficient [80], which quantifies how well each region fits within its assigned cluster relative to others, and the mean and standard deviation of the Adjusted Rand Index (ARI) [81], which assesses the reproducibility and stability of clustering results across bootstrap tests. Detailed definitions and formulas for these metrics are provided in the Non-negative Matrix Factorization of S1 Methods [80,81].

We set a Silhouette Coefficient threshold of 0.05, and *k* = 2–4 met this criterion (S6 Fig). Among these, *k* = 3 showed the highest reproducibility and stability (S6 Fig) and was therefore selected as our final solution. Regional loading coefficients for the three NMF components are shown in S7 Fig*.*

### Data harmonization

We applied the Combat-GAM [82] to harmonize data from the dHCP and BCP datasets, in order to compare the developmental patterns between the perinatal and postnatal periods. Specifically, we selected term-born neonates from dHCP (scan age 41–44 weeks) and infants from BCP (age 1–6 months) and assumed linear changes in regional SF coupling and whole-brain network metrics during the 0- to 6-month period. FD and gender were included as covariates, with the dHCP dataset set as the reference site. Notably, the harmonized data were used only for visualization of the developmental trajectory but not for quantitative analysis, due to the limited age overlap between the two datasets.

### Association with cognitive outcomes at 18 months

We evaluated the longitudinal associations between SF coupling at birth of term-born neonates and their neurodevelopmental outcomes at the 18-month follow-up within the dHCP dataset. A linear regression model was employed, where cluster-level SF coupling served as the independent variable after regressing out scan age, gender, and mean FD. The norm-referenced scores for cognition, motor, and language from Bayley Scales of Infant and Toddler Development, Third Edition (Bayley-Ⅲ) [83] were included as dependent variables. Family-related factors, including parental education levels and maternal mental health [84], were also included as covariates. Statistical significance was assessed using max-statistic permutation testing to control for multiple comparisons [85]. Specifically, in each of the 1,000 permutation iterations, we extracted the maximum absolute *t*-statistic of the coupling-behavior associations across all domains to construct an empirical null distribution. The *p*-values were then determined by comparing the real *t*-statistics against this null distribution. Given the dispersed timing of neuroimaging and cognitive assessments in BCP, prediction task was not performed for BCP.

## Results

### SF coupling at birth varies along the sensorimotor-association axis

**Fig 2:**
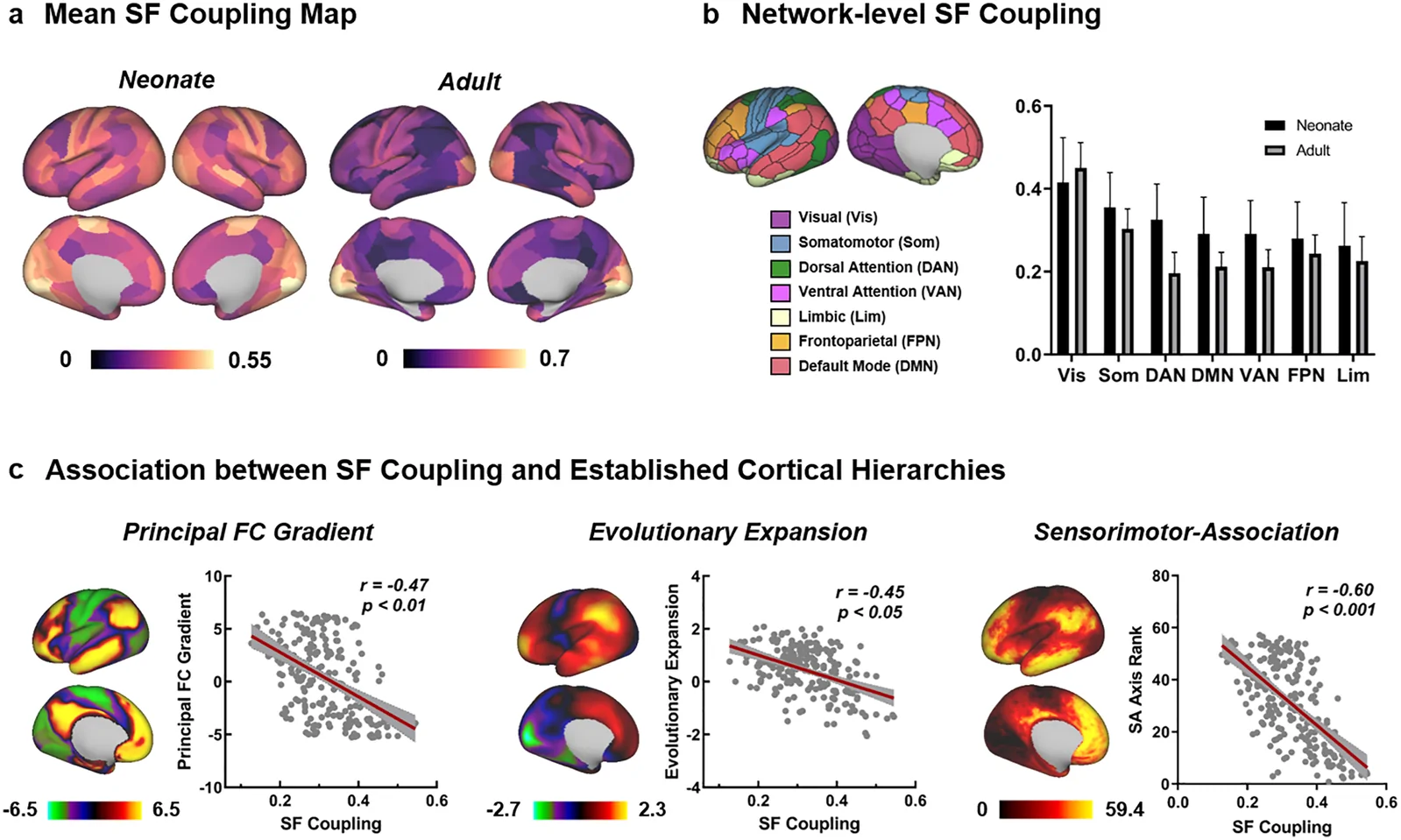
Spatial distribution pattern of SF coupling at birth. **(a)** Mean SF coupling maps for term-born neonates and young adults, obtained by averaging data from 376 term-born neonates in the dHCP dataset and 98 young adults in the HCP-YA dataset. **(b)** SF coupling strength across 7 sub- networks defined by Yeo’s atlas [76] in term-born neonates and young adults. **(c)** Correlation between the spatial pattern of neonatal SF coupling and established cortical hierarchies, including the principal functional gradient [26], cortical evolutionary expansion [73], and the sensorimotor-association axis [74]. The significance was evaluated using nonparametric spatial permutation testing. The underlying numerical data for this figure are provided in S1 Data.

As the first step, we established the spatial distribution of SF coupling at birth. A representative neonatal map was obtained by averaging results from 376 term-born neonates in the dHCP dataset (Fig 2a). We also generated an adult reference map by averaging data from 98 participants in the Human Connectome Project-Young Adult (HCP-YA) cohort (Fig 2a). Results showed that, compared with adults, the neonatal brain exhibited widespread higher coupling, except in the visual regions. After assigning cortical regions to sub-networks based on Yeo’s atlas [76], we identified significant differences in coupling strength across networks (ANOVA; neonates: *F* = 125.5, *p* < 0.0001; adults: *F* = 319.8, *p* < 0.0001). The highest coupling was observed in the visual and somatomotor networks in both groups, whereas the limbic and frontoparietal networks showed the lowest coupling in neonates but moderate coupling in adults (Fig 2b).

Furthermore, we examined whether the spatial pattern of SF coupling at birth aligned with established cortical hierarchies, including evolutionary surface area expansion derived from comparisons between human and macaque cortices [73], the principal FC gradient, which characterizes the functional axis from unimodal to transmodal cortex [26], and the sensorimotor-association (S-A) axis [74]. Significant negative correlations were observed between neonatal SF coupling and the FC gradient (*r* = −0.47, *p* = 0.003), evolutionary expansion (*r* = −0.45, *p* = 0.025), and the S-A axis (*r*=−0.60, *p* < 0.001), with all significance evaluated using spatial permutation tests (Fig 2c). These negative associations were in line with prior findings in adolescents and adults [22,23,28,29,40], as well as our adult results (S8 Fig). Collectively, these results suggest that SF coupling at birth already exhibits a hierarchical spatial pattern, characterized by progressive decoupling from primary sensorimotor regions to higher-order association areas [74], though in a relatively immature form compared with adults.

### Developmental trajectories of SF coupling exhibit spatiotemporal heterogeneity

**Fig 3:**
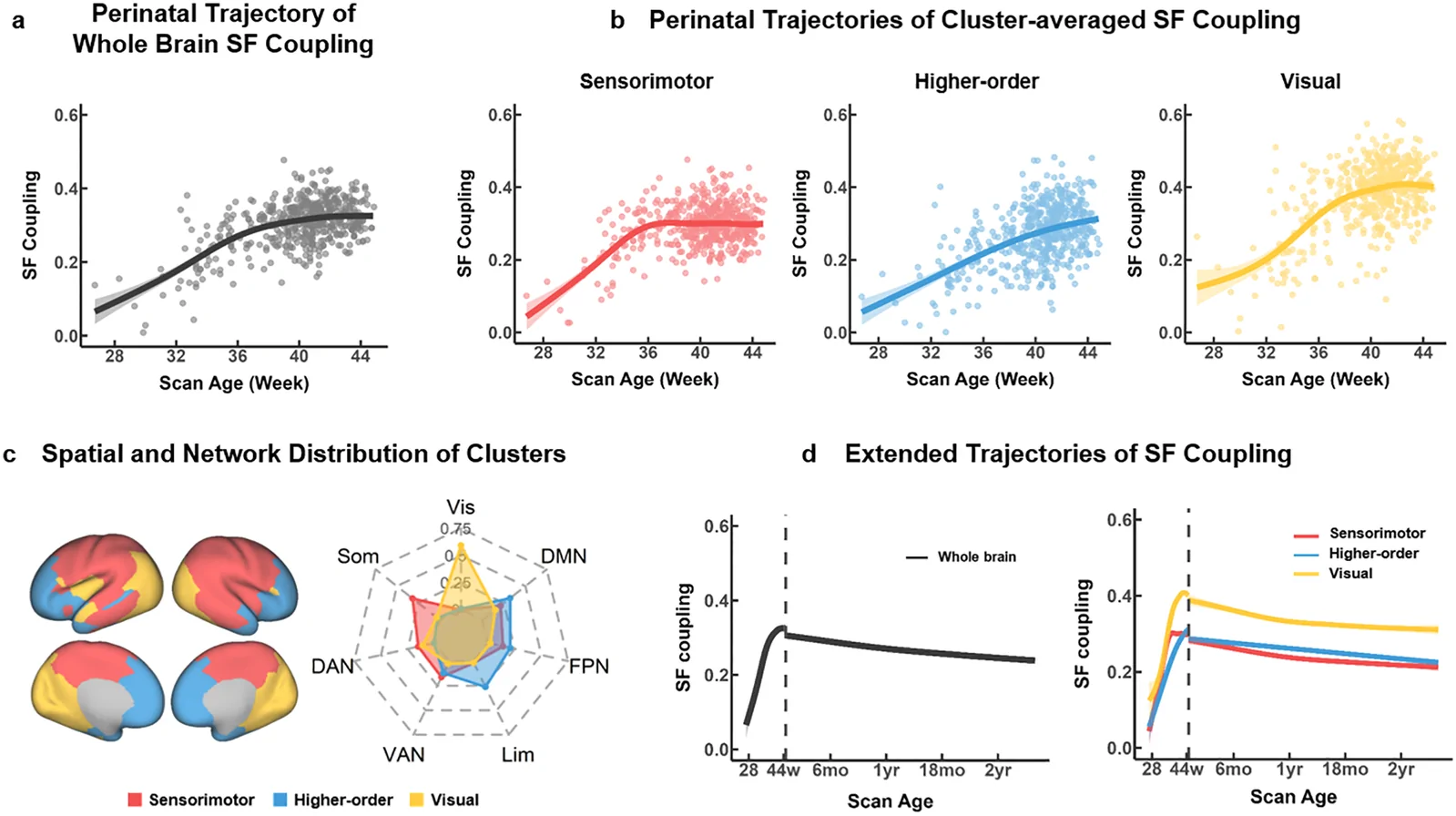
Developmental dynamics of SF coupling during the perinatal and postnatal period. **(a)** Perinatal trajectory of whole-brain SF coupling from 26 to 44 postmenstrual weeks, modeled using GAMM with age, gender, prematurity, and frame-wise displacement as fixed effects, and subject ID as random effect. **(b)** Perinatal trajectories of cluster-averaged SF coupling from 26 to 44 postmenstrual weeks, with the three clusters identified by NMF and trajectories modeled using GAMM with age, gender, prematurity, and frame-wise displacement as fixed effects, and subject ID as random effect. **(c)** Spatial distribution and radar chart of network distribution for the three NMF-derived clusters. Vis, Visual Network; Som, Somatomotor Network; DAN, Dorsal Attention Network; VAN, Ventral Attention Network; Lim, Limbic Network; FPN, Frontoparietal Network; DMN, Default Mode Network. **(d)** Extended trajectories of whole-brain and cluster-averaged SF coupling from 26 postmenstrual weeks to 28 postnatal months.

Using data from the dHCP and BCP cohorts, we depicted the developmental dynamics of SF coupling from the perinatal period through infancy and toddlerhood. During the perinatal period (26–44 gestational weeks), whole-brain SF coupling increased rapidly from 26 weeks onward, reached its fastest growth around 33 weeks, and then slowed down to a plateau near 42 weeks (Figs 3a and S9). To capture the spatial heterogeneity of SF coupling development, we applied NMF to regional SF coupling profiles in the dHCP cohort, partitioning the brain into several clusters. We identified three robust clusters, the sensorimotor, higher-order and visual cluster, which exhibited distinct perinatal trajectories. Coupling in the sensorimotor and visual clusters showed rapid growth, with the sensorimotor cluster reaching stabilization around 37 weeks and the visual cluster peaking at 42 weeks, whereas the higher-order cluster followed a slower and more prolonged increase extending to 44 weeks (Figs 3b and S10). The spatial and sub-network [76] distribution of the three clusters are shown in Fig 3c. The sensorimotor cluster occupies the central portion of the cortex and is primarily located in the somatomotor network. The higher-order cluster is anchored in the prefrontal and temporal pole area, with coverage across the DMN, limbic network, and FPN. The visual cluster is situated in the posterior cortex and predominantly overlaps with the visual network.

Using data from the BCP cohort, we extended the trajectories into the postnatal period (1–28 months). In contrast to the prenatal increase, whole-brain SF coupling showed a postnatal decrease (Fig 3d). This decline was consistently observed across clusters, with the sensorimotor and visual clusters showing a steeper decrease than the higher-order cluster during the first postnatal year (Figs 3d and S11). Together, we demonstrated that during early life, SF coupling followed a transitional trajectory, showing a rapid perinatal increase followed by a postnatal decline, with pronounced spatial heterogeneity.

### Functional maturation shapes the early development of SF coupling

**Fig 4:**
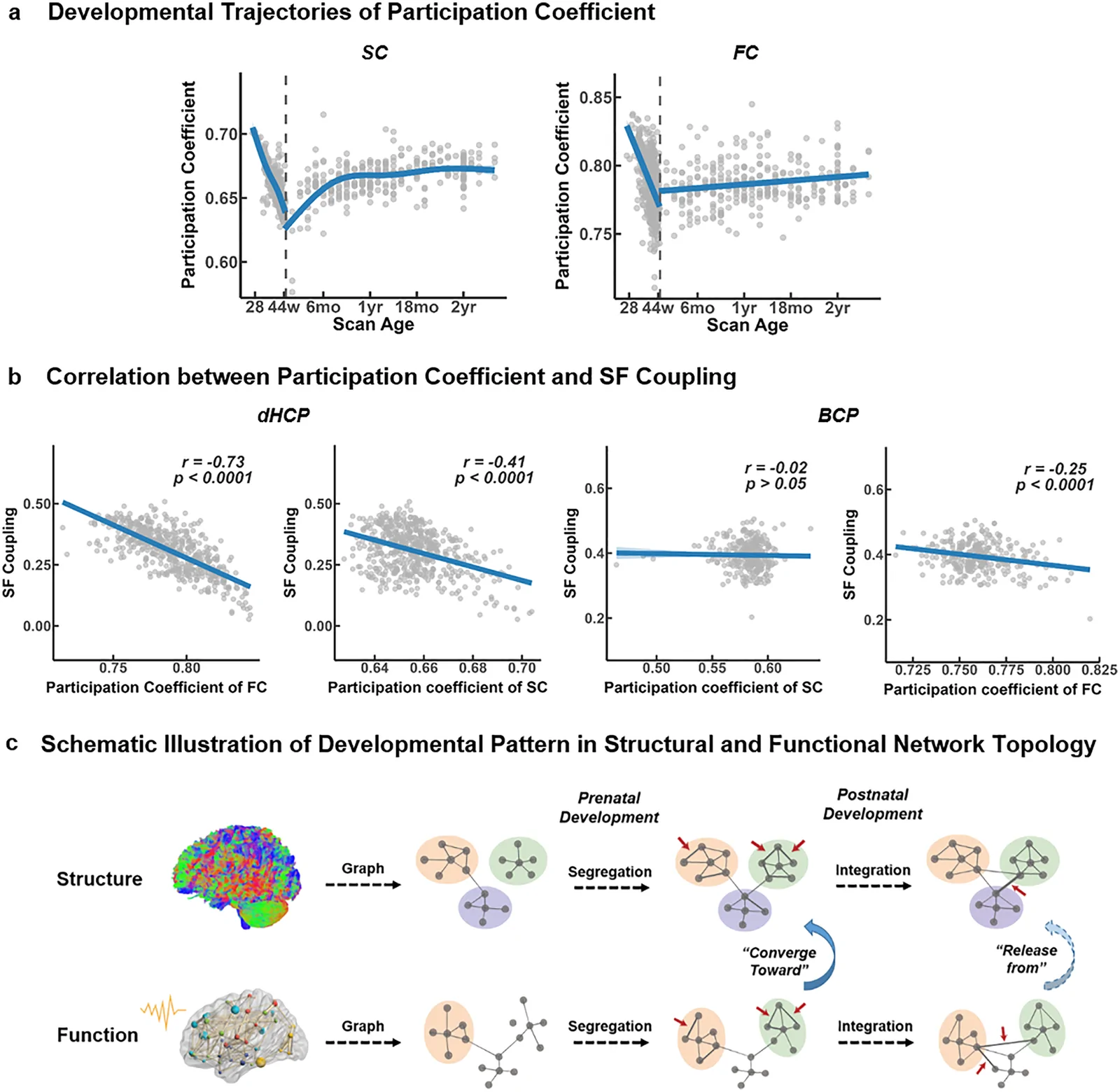
Development of network topology in structural and functional connectomes and its association with SF coupling. **(a)** Developmental trajectories of participation coefficients in structural (SC) and functional connectomes (FC), modeled using GAMM with gender, prematurity (dHCP only), frame-wise displacement (FC only), and protocol (BCP SC only) as fixed effects, and subject ID as a random effect. **(b)** Association between participation coefficients and SF coupling assessed by Pearson’s correlation; all p-values corrected for multiple comparisons using FDR. **(c)** Schematic illustration of developmental patterns in SC and FC network topology and the resulting dynamics of SF coupling across the perinatal and postnatal period.

Next, we aimed to identify the dominant contributor to the observed shift in SF coupling from the perinatal to postnatal period. To this end, we examined age-related changes in the network topology of structural connectome (SC) and functional connectome (FC) using two graph-theoretical metrics: participation coefficient and modularity. The participation coefficient quantifies the diversity of a node’s connections across modules, thereby reflecting the network’s capacity for global inter-module integration [71], while modularity measures the degree to which a network can be subdivided into distinct modules, indicating the extent of network segregation [86]. We observed that participation coefficients in both SC and FC decreased during the perinatal period (Fig 4a), whereas modularity increased with age (S12a Fig). In contrast, postnatal development followed an opposite trajectory: participation coefficients increased (Fig 4a) and modularity declined in both SC and FC (S12a Fig). The postnatal changes in SC were most pronounced during the first year of life and then plateaued, whereas FC exhibited a more gradual, linear developmental pattern. Together, these results indicated that perinatal development of both structure and functional networks was characterized by increasing segregation, upon entering the postnatal period, both of them shifted toward a more integrated topology (Fig 4c), consistent with a previous report [10].

To further explore how these topological changes associate with the development of SF coupling, we performed across-subject Pearson’s correlations between SF coupling and the two graph-theoretical metrics within each dataset. In the perinatal data from the dHCP cohort, participation coefficients in both SC and FC were significantly negatively correlated with SF coupling (Fig 4b). Notably, after controlling for SC participation coefficients, FC participation coefficients remained significantly associated with SF coupling (*p* < 0.0001), indicating an independent contribution of functional network to SF coupling. In the postnatal data from the BCP cohort, the negative correlation between FC participation coefficients and SF coupling remained significant, whereas the association with SC participation coefficients was no longer present. Regarding modularity, both SC and FC showed significant correlations with SF coupling during the perinatal period, but these associations were no longer evident across the postnatal windows (S12b Fig).

Collectively, these findings suggested that the development of SF coupling across the perinatal and postnatal periods was primarily accounted for by the maturation of functional connectivity. In other word, functional organization initially developed toward local anatomical architecture during the perinatal stage, and then decoupled from the anatomical constraints through global inter-module pathways postnatally (Fig 4c).

### SF coupling at birth is associated with later developmental outcomes

**Fig 5:**
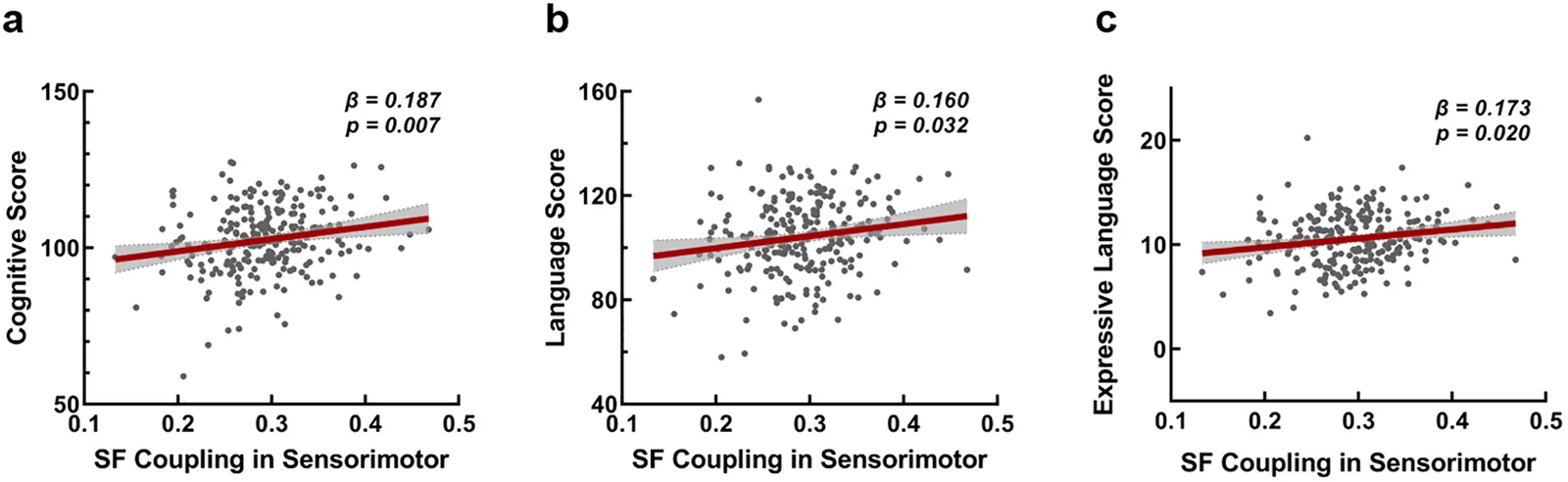
Association between SF coupling at birth and developmental outcomes at 18 months. **(a)** Stronger SF coupling in the sensorimotor cluster is associated with better cognitive outcomes. **(b)** Stronger SF coupling in the sensorimotor cluster is associated with better language outcomes. **(c)** Stronger SF coupling in the sensorimotor cluster is associated with better expressive language performance.

Moreover, we sought to investigate the clinical relevance of SF coupling by examining the association between SF coupling at birth (the transition point of the developmental trajectory) and later developmental outcomes. We included a standardized assessment scale, the Bayley-III, covering the cognitive, language and motor domains. These assessments were conducted at the 18-month follow-up visit from the dHCP. Out of 376 term-born subjects, 234 completed the Bayley-III and family socioeconomic assessment. Norm-referenced assessment scores were extracted for each domain and subdomain. Cluster-averaged SF coupling at birth was also calculated after regressing out the effects of age, gender, and head motion. Subsequently, the associations between SF coupling and assessment scores were examined using general linear models, adjusting for nuisance covariates including parental education and maternal postnatal mental health. Statistical significance was then determined via max-statistic permutation testing (1,000 iterations). As shown in Fig 5 and S1 Table, SF coupling in the sensorimotor cluster was significantly associated with both cognitive and language scores (cognitive, *β* = 0.187, 95% confidence interval = [0.061,0.313], *p*-permutation = 0.007; language, *β* = 0.160, 95% confidence interval = [0.033,0.287], *p*-permutation = 0.032) at 18 months, where stronger SF coupling linked to better outcome. Within the language subdomains, SF coupling in the sensorimotor cluster exhibited significant association with expressive language scores (*β* = 0.173, 95% confidence interval = [0.045,0.300], *p*-permutation = 0.020).

### Sensitivity analysis

To assess the robustness of our findings to the choice of structural connectivity matrix density, we repeated the primary analyses using alternative density of 20% and 30%, as well as the unthresholded matrix. Across all tested densities, the mean SF coupling map at birth consistently exhibited a spatial gradient along the sensorimotor–association cortical axis (S13–S15 Figs), although the statistical evidence was weaker in the unthresholded condition. During the perinatal period, SF coupling followed a similar rapid-increase-then-plateau trajectory across thresholded and unthresholded networks (S16 Fig). In addition, NMF revealed consistent clustering patterns, including sensorimotor, visual, and higher-order regions, as well as distinct developmental trajectories across these clusters (S17 Fig). Results from the BCP dataset further confirmed a consistent decreasing trend in SF coupling during the 1- to 28-month postnatal period across all tested densities (S18 Fig). Together, these findings indicate that the spatiotemporal developmental patterns of SF coupling are robust to the choice of structural connectivity matrix density.

## Discussion

In this study, we leveraged two large public datasets spanning both the perinatal and postnatal periods to characterize the spatiotemporal dynamics of SF coupling and its contributing factors in early life. SF coupling at birth exhibited a hierarchical spatial pattern that varied along the sensorimotor-association cortical axis. During the perinatal period, SF coupling increased steeply in a hierarchical manner and reached a plateau before birth. Entering the postnatal period, it shifted to a relatively slow decline during infancy and toddlerhood. These age-related changes in SF coupling were strongly associated with the development of functional connectivity, wherein functional organization first converges toward structural architecture prenatally and then gradually releases from anatomical constraints postnatally. Collectively, our findings offer important insights into how functional activity depends on anatomical architecture and how this relationship establishes and reconfigures during early life.

The SF relationship at birth exhibited a hierarchical variation from unimodal sensorimotor to transmodal association areas. This spatial pattern serves to balance local functional specialization with global integration. In sensorimotor regions, stronger SF coupling indicates that functional activity is largely shaped by underlying white matter pathways. In contrast, weaker coupling in association cortices reflects a relative release of functional organization from structural constraints, enabling more flexible signal integration via indirect and distributed pathways [87,88]. This hierarchical organization has been demonstrated in previous studies of adults [23,28], and our findings indicate that such a fundamental framework is already established following prenatal development.

SF coupling exhibited pronounced age-related increases during the perinatal period. By employing NMF, we revealed a multidimensional spatial hierarchy underlying perinatal SF coupling development. The principal axis of variation again reflected a unimodal-to-transmodal gradient, the principal functional gradient [26]. Growth curves indicated that unimodal cortices developed more rapidly and matured earlier than transmodal regions. Further, the visual cortex was dissociated from the sensorimotor cortices, showing a prolonged increasing course and higher coupling values at birth. This pattern reflects the sensorimotor-visual axis within the unimodal cortex, corresponding to a secondary gradient that has found in brain functional specialization [26] and gene expression profile [89].

While the rapid prenatal growth establishes the foundational framework of SF coupling at birth, the postnatal changes manifest as a developmental reconfiguration and refinement built upon this architecture. We observed a widespread decrease between 1 and 28 months, consistent with evidence from a prior study that used longitudinal data from children aged 1–2 years [41]. Similarly, a recent study of neonates aged 38–45 postmenstrual weeks reported decreased SF coupling during the postnatal period [42]. This reduction may extend up to approximately 7 years, as shown by another study [25]. Previous studies have found that average SF coupling increases during late childhood and adolescence [22,38,40], yet shows a general decrease across the life span [90], underscoring the complex and nonlinear nature of SF coupling development. Our study supports this view and establishes birth as an important transition point during early development.

A natural question that arises is what contributes to these developmental changes in SF coupling? Our findings offer compelling insights into this issue. We first showed that both structural and functional networks followed a developmental trajectory from perinatal segregation to segregation integration, in line with conclusions from a previous review of graph-theoretical modeling in baby brain networks [10]. A recent study utilizing the same datasets as our work also reported a similar trajectory in the early-life functional connectome, characterized by a shift from early segregation toward subsequent integration across both nodal and modular perspectives [19]. During the perinatal period, functional networks develop under the structural scaffolding and gradually become more aligned with it. This view is supported by prior research showing that brain structure develops earlier and reaches a relatively mature state by the time of birth [12,91–93]. Postnatally, structural networks rapidly strengthen inter-module connections during the first year of life [94,95], subsequently enter a phase of relative stability. Beyond structural maturation, the functional connectome undergoes more complex changes throughout the long postnatal period. During this time, specific regions gradually emerge as functional hubs that bridge distinct network modules [96]. Acting as relay stations in large-scale brain communication, these hubs enable indirect functional interactions between regions that do not share direct white-matter projections. For example, the basal ganglia and thalamus can serve as subcortical mediators linking the medial prefrontal cortex with motor areas [97], translating higher-order motivational or goal-related signals into concrete motor actions. This is evidenced by research showing that while thalamo-motor FC is relatively mature at birth, thalamo-DMN connectivity flourishes postnatally [98], providing a necessary ‘bridge’ for this circuit. In addition, the posterior parietal cortex functions as a transmodal hub that integrates spatial visual information and channels it toward motor systems for action planning and execution [99]. As these cross-modal communication pathways become stronger, the FC matrix becomes increasingly complex. Functional interactions are no longer restricted to direct anatomical pathways but can occur through multi-step network routes, which may ultimately manifest as the observed decline in SF coupling. Tooley and colleagues observed opposite developmental patterns of structural and functional network topology during the first postnatal month, accounting for the weakened coupling within this brief window [42]. We extended these trajectories over a longer time, from 26 postmenstrual weeks to 28 postnatal months, revealing distinct perinatal and postnatal patterns. More importantly, we identified a stronger association between functional networks maturation and SF coupling development.

In addition to charting normative trajectories, we highlighted the clinical relevance of SF coupling by establishing it as an early biomarker for individual developmental outcomes. Higher coupling in the sensorimotor cortex at birth was associated with better cognitive performance, suggesting that stronger SF tethering supports more efficient processing of environmental stimuli. The sensorimotor cluster also exhibited a robust association with language performance, particularly the expressive language performance, which may be attributable to its inclusion of Broca’s area, a core hub of the language network. Notably, while the sensorimotor cluster showed robust links to cognition and language, no significant association was observed for motor outcomes. This may be attributable to the earlier maturation of motor systems, which renders them more susceptible to the influence of environmental factors and postnatal experiences. While prior unimodal studies have demonstrated that network strength or topological metrics are correlated with cognitive and language development [19,100], our findings extend this evidence by proving that SF coupling similarly serves as a potent early marker of neurodevelopment.Together, these findings underscore the importance of SF coupling as a key developmental indicator, linking early-life brain organization to later developmental outcomes.

### Limitations of the study

Several limitations of the current study should be acknowledged. First, the limited age overlap between the two datasets constrains our ability to characterize continuous developmental trajectories across the perinatal-to-postnatal period. Future work leveraging longitudinal data that span the perinatal window will be crucial for capturing this developmental turning point. Additionally, although prematurity was taken into account as a covariate, preterm-born infants included in this study might not fully represent the typically developing population. Another point to note is that, in the brain-behavior analysis, we included only parental education level and maternal postnatal depression score as covariates. These measures do not fully capture broader aspects of socioeconomic status and postnatal family environment, which were not available in the current dataset.

The intrinsic limitations of diffusion MRI tractography should also be considered when interpreting our findings. Tracer-based validation studies have shown that diffusion MRI tractography has limited ability to recapitulate ground-truth white matter pathways: the correlation between tractography-derived cortical connectivity and tracer-derived anatomical connectivity in the non-human primate brain was only moderate (*r* = 0.59), and tractography accuracy decreased with increasing interareal path length [101]. This distance-dependent limitation is particularly relevant to the present study, because the perinatal and early postnatal periods are characterized by substantial brain growth and changing interareal distances, which may increase uncertainty in tractography estimates for long-range pathways across development and further affect estimates of SF coupling. These effects may be more pronounced in association cortices, as these regions support intermodule communication and are more likely to involve long-range structural connections. These potential influences should be considered when interpreting the development of SF coupling.

Relatedly, the true density of the human cortico-cortical connectome remains unknown because ground-truth cortical connectivity data are not available in humans. Non-human tracer-based studies provide useful context for this issue. A spatial embedding principle was demonstrated in macaque cortical networks, showing that connection probability declines exponentially with interareal distance [102]. Comparative work in rodents and primates suggests that cortical networks across species share this common principle of spatial embedding, and that the decay rate of the distance rule further varies systematically with brain volume [103–105]. These findings suggest that larger brains are likely to show a greater predominance of local connectivity and sparser long-range projections. Therefore, it is possible that the true human cortico-cortical connectome is relatively sparse, with a density substantially lower than the 25% threshold applied here. Further work using biologically informed density and distance constraints will be important for improving structural connectome reconstruction and refining SF coupling estimation.

Functional connectivity measurements are also susceptible to developmental and imaging-related factors. In particular, neurovascular coupling remains immature during fetal and early postnatal development. Because resting-state fMRI relies on the BOLD signal as an indirect proxy for neural activity, age-related changes in vascular reactivity, cerebral blood flow, oxygen metabolism, and neurovascular regulation may alter the relationship between neural activity and measured BOLD fluctuations. Therefore, developmental changes in FC should be interpreted in relation to both the maturation of neural functional organization and age-related changes in the physiological basis of the BOLD signal.

## Conclusions

In summary, this work elucidates the spatiotemporal dynamics of SF coupling from the perinatal period to toddlerhood and identifies the critical role of functional connectome in shaping the changes of the coupling. These findings advance our understanding of how anatomical basis scaffolds functional communication during early life and provide crucial insights into the organizational principles underlying typical brain development.

## Ethics statement

The dHCP study was approved by the United Kingdom Health Research Authority (Research Ethics Committee reference number: 14/LO/1169) and written parental consent was obtained in every case for imaging and open data release of the anonymized data. In the BCP, parents of all participants provided permission and written informed consent prior to participation, and all study procedures were approved by the University of North Carolina at Chapel Hill and the University of Minnesota Institutional Review Boards. For the HCP dataset, ethical approval was given by the Washington University Institutional Review Board (IRB #201204036).

## Supporting information

S1 Methods Additional methodological details, including image preprocessing, cortical parcellation registration, network metric definitions and computations, non-negative matrix factorization, and developmental trajectory modeling. (DOCX)

S1 Data Supporting data underlying Figs 2B, S3, S4, S5B, S13B, S14B, and S15B. (XLSX)

S1 Fig Flowchart of data inclusion and exclusion. **(a)** The dHCP dataset. **(b)** The BCP dataset.
(TIF)

S2 Fig Longitudinal data distribution of subjects in two datasets. (a) The dHCP dataset. **(b)** The BCP dataset.
(TIF)

S3 Fig Differences in SF coupling between the two BCP dMRI protocols. Differences in whole-brain **(a)** and cluster-averaged **(b)** SF coupling between the two BCP dMRI protocols. The y-axis represents residual after regressing out of age, gender, frame-wise displacement, and subject random effect. The underlying numerical data for this figure are provided in S1 Data.
(TIF)

S4 Fig Differences in network metrics between the two BCP dMRI protocols. Differences in participation coefficient (PC, **a**) and modularity **(b)** of structural connectome (SC) between the two BCP dMRI protocols. The y-axis represents residual after regressing out of age, gender, and subject random effect. The underlying numerical data for this figure are provided in S1 Data.
(TIF)

S5 Fig Sensitivity analysis of FOD estimation methods in a BCP subsample (*N* = 50). **(a)** Scatter plot showing a strong linear correlation between coupling values derived from MT-CSD and ST-CSD. **(b)** Boxplots showing that absolute coupling values do not differ significantly between the two pipelines (ns: not significant). **(c)** Developmental trajectories derived from the ST-CSD pipeline capture the same postnatal decline in coupling as observed with MT-CSD, demonstrating the robustness of our results. The underlying numerical data for this figure are provided in S1 Data.
(TIF)

S6 Fig Evaluation metrics of NMF solution. Silhouette coefficient **(a)**, mean of adjusted rand index (ARI, **b**), and standard deviation of adjusted rand index **(c)** of NMF solutions at multiple resolutions ranging from 2 to 10.
(TIF)

S7 Fig Regional loading coefficients for each NMF component. (TIF)

S8 Fig Correlation between the spatial pattern of adult SF coupling and established cortical hierarchies. The significance was evaluated using nonparametric spatial permutation testing.
(TIF)

S9 Fig The change rate (first-order derivative of the developmental trajectory) for whole-brain SF coupling from 26 to 44 postmenstrual weeks. (TIF)

S10 Fig The change rate (first-order derivative of the developmental trajectory) for cluster-averaged SF coupling from 26 to 44 gestational weeks. (TIF)

S11 Fig The change rate (first-order derivative of the developmental trajectory) for cluster-averaged SF coupling from 1 to 28 postnatal months. (TIF)

S12 Fig Development of modularity in structural and functional connectomes and its association with SF coupling. **(a)** Developmental trajectories of modularity in structural (SC) and functional connectomes (FC), modeled using GAMMs with gender, prematurity (dHCP only), frame-wise displacement (FC only), and protocol (BCP SC only) as fixed effects, and subject ID as random effect. **(b)** Association between modularity and SF coupling assessed using Pearson’s correlation; all p-values were corrected for multiple comparisons using FDR.
(TIF)

S13 Fig Spatial distribution of SF coupling at birth using an unthresholded structural connectivity matrix density. **(a)** Mean SF coupling map for term-born neonates, obtained by averaging data from 376 term-born neonates in the dHCP dataset. **(b)** SF coupling strength across 7 sub-networks defined by Yeo’s atlas in term-born neonates. **(c)** Correlation between the spatial pattern of neonatal SF coupling and established cortical hierarchies. The significance was evaluated using nonparametric spatial permutation testing. The underlying numerical data for this figure are provided in S1 Data.
(TIF)

S14 Fig Spatial distribution of SF coupling at birth using a 20% structural connectivity matrix density. **(a)** Mean SF coupling map for term-born neonates, obtained by averaging data from 376 term-born neonates in the dHCP dataset. **(b)** SF coupling strength across 7 sub-networks defined by Yeo’s atlas in term-born neonates. **(c)** Correlation between the spatial pattern of neonatal SF coupling and established cortical hierarchies. The significance was evaluated using nonparametric spatial permutation testing. The underlying numerical data for this figure are provided in S1 Data.
(TIF)

S15 Fig Spatial distribution of SF coupling at birth using a 30% structural connectivity matrix density. **(a)** Mean SF coupling map for term-born neonates, obtained by averaging data from 376 term-born neonates in the dHCP dataset. **(b)** SF coupling strength across 7 sub-networks defined by Yeo’s atlas in term-born neonates. **(c)** Correlation between the spatial pattern of neonatal SF coupling and established cortical hierarchies. The significance was evaluated using nonparametric spatial permutation testing. The underlying numerical data for this figure are provided in S1 Data.
(TIF)

S16 Fig Perinatal trajectories of whole-brain SF coupling from 26 to 44 postmenstrual weeks across structural connectivity matrix densities of 20%, 25%, 30%, and the unthresholded (raw) matrix. (TIF)

S17 Fig NMF-derived clusters and their trajectories across structural connectivity matrix densities of 20%, 25%, 30%, and the unthresholded (raw) matrix. **(a)** Spatial distribution of NMF-derived clusters at the three structural connectivity matrix densities. **(b)** Perinatal trajectories of cluster-averaged SF coupling from 26 to 44 postmenstrual weeks across the three densities.
(TIF)

S18 Fig Postnatal trajectories of whole-brain SF coupling from 1 to 28 months across structural connectivity matrix densities of 20%, 25%, 30%, and the unthresholded (raw) matrix. (TIF)

S1 Table Association between SF coupling and developmental outcomes at 18 months. (DOCX)

## Abbreviations

ACT: Anatomically-Constrained Tractography
ARI: Adjusted Rand Index
BCP: Baby Connectome Project
dHCP: developing Human Connectome Project
dMRI: diffusion MRI
DMN: default mode network
EPI: echo-planar imaging
FC: functional connectome
FD: frame-wise displacement
FODs: fiber orientation distributions
GA: gestational age
HCP-YA: Human Connectome Project-Young Adult
MB: multiband
MRI: magnetic resonance imaging
MSMT-CSD: multi-shell multi-tissue constrained spherical deconvolution
NMF: non-negative matrix factorization
rs-fMRI: resting-state functional MRI
SC: structural connectome
SF: structure–function
ST-CSD: single-tissue constrained spherical deconvolution
TE: echo time
TEA: term-equivalent age
TR: repetition time
3D-MPRAGE: 3D magnetization-prepared rapid gradient echo

## References

[1] Kostović I, Sedmak G, Judaš M. Neural histology and neurogenesis of the human fetal and infant brain. Neuroimage. 2019;188:743–73. doi: 10.1016/j.neuroimage.2018.12.043 30594683

[2] Ouyang M, Dubois J, Yu Q, Mukherjee P, Huang H. Delineation of early brain development from fetuses to infants with diffusion MRI and beyond. Neuroimage. 2019;185:836–50. doi: 10.1016/j.neuroimage.2018.04.017 29655938 PMC6185831

[3] Stiles J, Jernigan TL. The basics of brain development. Neuropsychol Rev. 2010;20(4):327–48. doi: 10.1007/s11065-010-9148-4 21042938 PMC2989000

[4] Qiu A, Mori S, Miller MI. Diffusion tensor imaging for understanding brain development in early life. Annu Rev Psychol. 2015;66:853–76. doi: 10.1146/annurev-psych-010814-015340 25559117 PMC4474038

[5] Huang H, Zhang J, Wakana S, Zhang W, Ren T, Richards LJ, et al. White and gray matter development in human fetal, newborn and pediatric brains. Neuroimage. 2006;33(1):27–38. doi: 10.1016/j.neuroimage.2006.06.009 16905335

[6] Turk E, van den Heuvel MI, Benders MJ, de Heus R, Franx A, Manning JH, et al. Functional connectome of the fetal brain. J Neurosci. 2019;39(49):9716–24. doi: 10.1523/JNEUROSCI.2891-18.2019 31685648 PMC6891066

[7] Cao M, He Y, Dai Z, Liao X, Jeon T, Ouyang M, et al. Early development of functional network segregation revealed by connectomic analysis of the preterm human brain. Cereb Cortex. 2017;27(3):1949–63. doi: 10.1093/cercor/bhw038 26941380 PMC6059235

[8] Bassett DS, Sporns O. Network neuroscience. Nat Neurosci. 2017;20(3):353–64. doi: 10.1038/nn.4502 28230844 PMC5485642

[9] Cao M, Huang H, He Y. Developmental connectomics from infancy through early childhood. Trends Neurosci. 2017;40(8):494–506. doi: 10.1016/j.tins.2017.06.003 28684174 PMC5975640

[10] Zhao T, Xu Y, He Y. Graph theoretical modeling of baby brain networks. Neuroimage. 2019;185:711–27. doi: 10.1016/j.neuroimage.2018.06.038 29906633

[11] Takahashi E, Folkerth RD, Galaburda AM, Grant PE. Emerging cerebral connectivity in the human fetal brain: an MR tractography study. Cereb Cortex. 2012;22(2):455–64. doi: 10.1093/cercor/bhr126 21670100 PMC3256410

[12] Ball G, Aljabar P, Zebari S, Tusor N, Arichi T, Merchant N, et al. Rich-club organization of the newborn human brain. Proc Natl Acad Sci U S A. 2014;111(20):7456–61. doi: 10.1073/pnas.1324118111 24799693 PMC4034228

[13] Huang H, Shu N, Mishra V, Jeon T, Chalak L, Wang ZJ, et al. Development of human brain structural networks through infancy and childhood. Cereb Cortex. 2015;25(5):1389–404. doi: 10.1093/cercor/bht335 24335033 PMC4397575

[14] Cao M, Huang H, Peng Y, Dong Q, He Y. Toward developmental connectomics of the human brain. Front Neuroanat. 2016;10(25). doi: 10.3389/fnana.2016.00025 PMC4814555 27064378

[15] Eyre M, Fitzgibbon SP, Ciarrusta J, Cordero-Grande L, Price AN, Poppe T, et al. The Developing Human Connectome Project: typical and disrupted perinatal functional connectivity. Brain. 2021;144(7):2199–213. doi: 10.1093/brain/awab118 33734321 PMC8370420

[16] Gao W, Zhu H, Giovanello KS, Smith JK, Shen D, Gilmore JH, et al. Evidence on the emergence of the brain’s default network from 2-week-old to 2-year-old healthy pediatric subjects. Proc Natl Acad Sci U S A. 2009;106(16):6790–5. doi: 10.1073/pnas.0811221106 19351894 PMC2672537

[17] Gao W, Alcauter S, Smith JK, Gilmore JH, Lin W. Development of human brain cortical network architecture during infancy. Brain Struct Funct. 2015;220(2):1173–86. doi: 10.1007/s00429-014-0710-3 24469153 PMC4850360

[18] Yin W, Li T, Wu Z, Hung S-C, Hu D, Gui Y, et al. Charting brain functional development from birth to 6 years of age. Nat Hum Behav. 2025;9(6):1246–59. doi: 10.1038/s41562-025-02160-2 40234630 PMC12185323

[19] Li Q, Xia M, Zeng D, Xu Y, Sun L, Liang X, et al. Development of segregation and integration of functional connectomes during the first 1,000 days. Cell Rep. 2024;43(5). doi: 10.1016/j.celrep.2024.114168 38700981

[20] Fotiadis P, Parkes L, Davis KA, Satterthwaite TD, Shinohara RT, Bassett DS. Structure-function coupling in macroscale human brain networks. Nat Rev Neurosci. 2024;25(10):688–704. doi: 10.1038/s41583-024-00846-6 39103609

[21] Gu Z, Jamison KW, Sabuncu MR, Kuceyeski A. Heritability and interindividual variability of regional structure-function coupling. Nat Commun. 2021;12(1):4894. doi: 10.1038/s41467-021-25184-4 34385454 PMC8361191

[22] Baum GL, Cui Z, Roalf DR, Ciric R, Betzel RF, Larsen B, et al. Development of structure-function coupling in human brain networks during youth. Proc Natl Acad Sci U S A. 2020;117(1):771–8. doi: 10.1073/pnas.1912034117 31874926 PMC6955327

[23] Fotiadis P, Cieslak M, He X, Caciagli L, Ouellet M, Satterthwaite TD, et al. Myelination and excitation-inhibition balance synergistically shape structure-function coupling across the human cortex. Nat Commun. 2023;14(1):6115. doi: 10.1038/s41467-023-41686-9 37777569 PMC10542365

[24] Chan SY, Ong ZY, Ngoh ZM, Chong YS, Zhou JH, Fortier MV, et al. Structure-function coupling within the reward network in preschool children predicts executive functioning in later childhood. Dev Cogn Neurosci. 2022;55:101107. doi: 10.1016/j.dcn.2022.101107 35413663 PMC9010704

[25] Chan SY, Ngoh ZM, Ong ZY, Teh AL, Kee MZL, Zhou JH, et al. The influence of early-life adversity on the coupling of structural and functional brain connectivity across childhood. Nat Mental Health. 2024;2(1):52–62. doi: 10.1038/s44220-023-00162-5

[26] Margulies DS, Ghosh SS, Goulas A, Falkiewicz M, Huntenburg JM, Langs G, et al. Situating the default-mode network along a principal gradient of macroscale cortical organization. Proc Natl Acad Sci U S A. 2016;113(44):12574–9. doi: 10.1073/pnas.1608282113 27791099 PMC5098630

[27] von Economo CF, Koskinas GN. Die cytoarchitektonik der hirnrinde des erwachsenen menschen. Arch NeurPsych. 1926;16(6):816. doi: 10.1001/archneurpsyc.1926.02200300136013

[28] Vázquez-Rodríguez B, Suárez LE, Markello RD, Shafiei G, Paquola C, Hagmann P, et al. Gradients of structure-function tethering across neocortex. Proc Natl Acad Sci U S A. 2019;116(42):21219–27. doi: 10.1073/pnas.1903403116 31570622 PMC6800358

[29] Preti MG, Van De Ville D. Decoupling of brain function from structure reveals regional behavioral specialization in humans. Nat Commun. 2019;10(1):4747. doi: 10.1038/s41467-019-12765-7 31628329 PMC6800438

[30] Popp JL, Thiele JA, Faskowitz J, Seguin C, Sporns O, Hilger K. Structural-functional brain network coupling predicts human cognitive ability. Neuroimage. 2024;290:120563. doi: 10.1016/j.neuroimage.2024.120563 38492685

[31] Medaglia JD, Huang W, Karuza EA, Kelkar A, Thompson-Schill SL, Ribeiro A, et al. Functional alignment with anatomical networks is associated with cognitive flexibility. Nat Hum Behav. 2018;2(2):156–64. doi: 10.1038/s41562-017-0260-9 30498789 PMC6258039

[32] Griffa A, Amico E, Liégeois R, Van De Ville D, Preti MG. Brain structure-function coupling provides signatures for task decoding and individual fingerprinting. Neuroimage. 2022;250:118970. doi: 10.1016/j.neuroimage.2022.118970 35124226

[33] Wang J, Khosrowabadi R, Ng KK, Hong Z, Chong JSX, Wang Y, et al. Alterations in brain network topology and structural-functional connectome coupling relate to cognitive impairment. Front Aging Neurosci. 2018;10:404. doi: 10.3389/fnagi.2018.00404 30618711 PMC6300727

[34] Zarkali A, McColgan P, Leyland L-A, Lees AJ, Rees G, Weil RS. Organisational and neuromodulatory underpinnings of structural-functional connectivity decoupling in patients with Parkinson’s disease. Commun Biol. 2021;4(1):86. doi: 10.1038/s42003-020-01622-9 33469150 PMC7815846

[35] Chen H, Geng W, Shang S, Shi M, Zhou L, Jiang L, et al. Alterations of brain network topology and structural connectivity-functional connectivity coupling in capsular versus pontine stroke. Eur J Neurol. 2021;28(6):1967–76. doi: 10.1111/ene.14794 33657258

[36] Collin G, Scholtens LH, Kahn RS, Hillegers MHJ, van den Heuvel MP. Affected anatomical rich club and structural-functional coupling in young offspring of schizophrenia and bipolar disorder patients. Biol Psychiatry. 2017;82(10):746–55. doi: 10.1016/j.biopsych.2017.06.013 28734460

[37] Chu T, Si X, Xie H, Ma H, Shi Y, Yao W, et al. Regional structural-functional connectivity coupling in major depressive disorder is associated with neurotransmitter and genetic profiles. Biol Psychiatry. 2025;97(3):290–301. doi: 10.1016/j.biopsych.2024.08.022 39218135

[38] Soman SM, Vijayakumar N, Thomson P, Ball G, Hyde C, Silk TJ. Cortical structural and functional coupling during development and implications for attention deficit hyperactivity disorder. Transl Psychiatry. 2023;13(1). doi: 10.1038/s41398-023-02546-8 PMC10336084 37433763

[39] Xu M, Li X, Teng T, Huang Y, Liu M, Long Y, et al. Reconfiguration of structural and functional connectivity coupling in patient subgroups with adolescent depression. JAMA Netw Open. 2024;7(3):e241933. doi: 10.1001/jamanetworkopen.2024.1933 38470418 PMC10933730

[40] Feng G, Wang Y, Huang W, Chen H, Cheng J, Shu N. Spatial and temporal pattern of structure-function coupling of human brain connectome with development. Elife. 2024;13:RP93325. doi: 10.7554/eLife.93325 38900563 PMC11189631

[41] Hong Y, Cornea E, Girault JB, Bagonis M, Foster M, Kim SH, et al. Structural and functional connectome relationships in early childhood. Dev Cogn Neurosci. 2023;64:101314. doi: 10.1016/j.dcn.2023.101314 37898019 PMC10630618

[42] Tooley UA, Kenley JK, Camacho MC, Latham A, Alexopoulos D, Nielsen AN, et al. Structure-function coupling in the first month of life: associations with age and attention. Proc Natl Acad Sci U S A. 2025;122(23):e2412729122. doi: 10.1073/pnas.2412729122 40455980 PMC12168018

[43] Wang R, Fang T, Zhang Y, Cheng Y, Wang C, Chen Y, et al. The overgrowth of structure-function coupling in premature brain during infancy. Dev Cogn Neurosci. 2025;72:101535. doi: 10.1016/j.dcn.2025.101535 40020404 PMC11910680

[44] Mao W, Chen Y, He Z, Wang Z, Xiao Z, Sun Y, et al. Brain structural connectivity guided vision transformers for identification of functional connectivity characteristics in preterm neonates. IEEE J Biomed Health Inform. 2024;28(4):2223–34. doi: 10.1109/JBHI.2024.3355020 38285570

[45] Howell BR, Styner MA, Gao W, Yap P-T, Wang L, Baluyot K, et al. The UNC/UMN Baby Connectome Project (BCP): an overview of the study design and protocol development. Neuroimage. 2019;185:891–905. doi: 10.1016/j.neuroimage.2018.03.049 29578031 PMC6151176

[46] Van Essen DC, Smith SM, Barch DM, Behrens TEJ, Yacoub E, Ugurbil K, et al. The WU-Minn Human Connectome Project: an overview. Neuroimage. 2013;80:62–79. doi: 10.1016/j.neuroimage.2013.05.041 23684880 PMC3724347

[47] Edwards AD, Rueckert D, Smith SM, Abo Seada S, Alansary A, Almalbis J, et al. The developing Human Connectome Project neonatal data release. Frontiers in Neuroscience. 2022;16. doi: 10.3389/fnins.2022.886772 PMC9169090 35677357

[48] Glasser MF, Sotiropoulos SN, Wilson JA, Coalson TS, Fischl B, Andersson JL, et al. The minimal preprocessing pipelines for the Human Connectome Project. Neuroimage. 2013;80:105–24. doi: 10.1016/j.neuroimage.2013.04.127 23668970 PMC3720813

[49] Smith SM, Beckmann CF, Andersson J, Auerbach EJ, Bijsterbosch J, Douaud G, et al. Resting-state fMRI in the Human Connectome Project. Neuroimage. 2013;80:144–68. doi: 10.1016/j.neuroimage.2013.05.039 23702415 PMC3720828

[50] Sotiropoulos SN, Jbabdi S, Xu J, Andersson JL, Moeller S, Auerbach EJ, et al. Advances in diffusion MRI acquisition and processing in the Human Connectome Project. Neuroimage. 2013;80:125–43. doi: 10.1016/j.neuroimage.2013.05.057 23702418 PMC3720790

[51] Bastiani M, Andersson JLR, Cordero-Grande L, Murgasova M, Hutter J, Price AN, et al. Automated processing pipeline for neonatal diffusion MRI in the developing Human Connectome Project. Neuroimage. 2019;185:750–63. doi: 10.1016/j.neuroimage.2018.05.064 29852283 PMC6299258

[52] Fitzgibbon SP, Harrison SJ, Jenkinson M, Baxter L, Robinson EC, Bastiani M, et al. The developing Human Connectome Project (dHCP) automated resting-state functional processing framework for newborn infants. Neuroimage. 2020;223:117303. doi: 10.1016/j.neuroimage.2020.117303 32866666 PMC7762845

[53] Makropoulos A, Robinson EC, Schuh A, Wright R, Fitzgibbon S, Bozek J, et al. The developing human connectome project: a minimal processing pipeline for neonatal cortical surface reconstruction. Neuroimage. 2018;173:88–112. doi: 10.1016/j.neuroimage.2018.01.054 29409960 PMC6783314

[54] Yan CG, Wang XD, Zuo XN, Zang YF. DPABI: data processing & analysis for (resting-state) brain imaging. Neuroinformatics. 2016;14(3):339–51. doi: 10.1007/s12021-016-9299-4 27075850

[55] Tournier J-D, Calamante F, Connelly A. Robust determination of the fibre orientation distribution in diffusion MRI: non-negativity constrained super-resolved spherical deconvolution. Neuroimage. 2007;35(4):1459–72. doi: 10.1016/j.neuroimage.2007.02.016 17379540

[56] Tournier JD, Calamante F, Connelly A. Improved probabilistic streamlines tractography by 2nd order integration over fibre orientation distributions. Proc Int Soc Magn Reson Med. 2010;1670.

[57] Smith RE, Tournier J-D, Calamante F, Connelly A. Anatomically-constrained tractography: improved diffusion MRI streamlines tractography through effective use of anatomical information. Neuroimage. 2012;62(3):1924–38. doi: 10.1016/j.neuroimage.2012.06.005 22705374

[58] Smith RE, Tournier J-D, Calamante F, Connelly A. SIFT: spherical-deconvolution informed filtering of tractograms. Neuroimage. 2013;67:298–312. doi: 10.1016/j.neuroimage.2012.11.049 23238430

[59] Wang L, Wu Z, Chen L, Sun Y, Lin W, Li G. iBEAT V2.0: a multisite-applicable, deep learning-based pipeline for infant cerebral cortical surface reconstruction. Nat Protoc. 2023;18(5):1488–509. doi: 10.1038/s41596-023-00806-x 36869216 PMC10241227

[60] Zhang H, Shen D, Lin W. Resting-state functional MRI studies on infant brains: a decade of gap-filling efforts. Neuroimage. 2019;185:664–84. doi: 10.1016/j.neuroimage.2018.07.004 29990581 PMC6289773

[61] Glasser MF, Smith SM, Marcus DS, Andersson JLR, Auerbach EJ, Behrens TEJ, et al. The Human Connectome Project’s neuroimaging approach. Nat Neurosci. 2016;19(9):1175–87. doi: 10.1038/nn.4361 27571196 PMC6172654

[62] Liu T, Wu J, Zhao Z, Li M, Lv Y, Li M, et al. Developmental pattern of association fibers and their interaction with associated cortical microstructures in 0-5-month-old infants. Neuroimage. 2022;261:119525. doi: 10.1016/j.neuroimage.2022.119525 35908606

[63] Jeurissen B, Tournier J-D, Dhollander T, Connelly A, Sijbers J. Multi-tissue constrained spherical deconvolution for improved analysis of multi-shell diffusion MRI data. Neuroimage. 2014;103:411–26. doi: 10.1016/j.neuroimage.2014.07.061 25109526

[64] Li M, Xu X, Cao Z, Chen R, Zhao R, Zhao Z, et al. Multi-modal multi-resolution atlas of the human neonatal cerebral cortex based on microstructural similarity. Neuroimage. 2023;272:120071. doi: 10.1016/j.neuroimage.2023.120071 37003446

[65] Bozek J, Makropoulos A, Schuh A, Fitzgibbon S, Wright R, Glasser MF, et al. Construction of a neonatal cortical surface atlas using Multimodal Surface Matching in the Developing Human Connectome Project. Neuroimage. 2018;179:11–29. doi: 10.1016/j.neuroimage.2018.06.018 29890325 PMC6783315

[66] Williams LZJ, Fitzgibbon SP, Bozek J, Winkler AM, Dimitrova R, Poppe T, et al. Structural and functional asymmetry of the neonatal cerebral cortex. Nat Hum Behav. 2023;7(6):942–55. doi: 10.1038/s41562-023-01542-8 36928781 PMC7618908

[67] Chen L, Wu Z, Hu D, Wang Y, Zhao F, Zhong T, et al. A 4D infant brain volumetric atlas based on the UNC/UMN baby connectome project (BCP) cohort. Neuroimage. 2022;253:119097. doi: 10.1016/j.neuroimage.2022.119097 35301130 PMC9155180

[68] Robinson EC, Jbabdi S, Glasser MF, Andersson J, Burgess GC, Harms MP, et al. MSM: a new flexible framework for Multimodal Surface Matching. Neuroimage. 2014;100:414–26. doi: 10.1016/j.neuroimage.2014.05.069 24939340 PMC4190319

[69] Avants BB, Epstein CL, Grossman M, Gee JC. Symmetric diffeomorphic image registration with cross-correlation: evaluating automated labeling of elderly and neurodegenerative brain. Med Image Anal. 2008;12(1):26–41. doi: 10.1016/j.media.2007.06.004 17659998 PMC2276735

[70] Rubinov M, Sporns O. Complex network measures of brain connectivity: uses and interpretations. Neuroimage. 2010;52(3):1059–69. doi: 10.1016/j.neuroimage.2009.10.003 19819337

[71] Guimerà R, Amaral LAN. Cartography of complex networks: modules and universal roles. J Stat Mech. 2005;2005(P02001):nihpa35573. doi: 10.1088/1742-5468/2005/02/P02001 18159217 PMC2151742

[72] Newman MEJ. Modularity and community structure in networks. Proc Natl Acad Sci U S A. 2006;103(23):8577–82. doi: 10.1073/pnas.0601602103 16723398 PMC1482622

[73] Hill J, Inder T, Neil J, Dierker D, Harwell J, Van Essen D. Similar patterns of cortical expansion during human development and evolution. Proc Natl Acad Sci U S A. 2010;107(29):13135–40. doi: 10.1073/pnas.1001229107 20624964 PMC2919958

[74] Sydnor VJ, Larsen B, Bassett DS, Alexander-Bloch A, Fair DA, Liston C, et al. Neurodevelopment of the association cortices: patterns, mechanisms, and implications for psychopathology. Neuron. 2021;109(18):2820–46. doi: 10.1016/j.neuron.2021.06.016 34270921 PMC8448958

[75] Alexander-Bloch AF, Shou H, Liu S, Satterthwaite TD, Glahn DC, Shinohara RT, et al. On testing for spatial correspondence between maps of human brain structure and function. Neuroimage. 2018;178:540–51. doi: 10.1016/j.neuroimage.2018.05.070 29860082 PMC6095687

[76] Yeo BTT, Krienen FM, Sepulcre J, Sabuncu MR, Lashkari D, Hollinshead M, et al. The organization of the human cerebral cortex estimated by intrinsic functional connectivity. J Neurophysiol. 2011;106(3):1125–65. doi: 10.1152/jn.00338.2011 21653723 PMC3174820

[77] Wang F, Lian C, Wu Z, Zhang H, Li T, Meng Y, et al. Developmental topography of cortical thickness during infancy. Proc Natl Acad Sci U S A. 2019;116(32):15855–60. doi: 10.1073/pnas.1821523116 31332010 PMC6689940

[78] Huang Y, Wu Z, Wang F, Hu D, Li T, Guo L, et al. Mapping developmental regionalization and patterns of cortical surface area from 29 post-menstrual weeks to 2 years of age. Proc Natl Acad Sci U S A. 2022;119(33):e2121748119. doi: 10.1073/pnas.2121748119 35939665 PMC9388141

[79] Nazeri A, Krsnik Ž, Kostović I, Ha SM, Kopić J, Alexopoulos D, et al. Neurodevelopmental patterns of early postnatal white matter maturation represent distinct underlying microstructure and histology. Neuron. 2022;110(23):4015-4030.e4. doi: 10.1016/j.neuron.2022.09.020 36243003 PMC9742299

[80] Rousseeuw PJ. Silhouettes: a graphical aid to the interpretation and validation of cluster analysis. J Comput Appl Math. 1987;20:53–65. doi: 10.1016/0377-0427(87)90125-7

[81] Hubert L, Arabie P. Comparing partitions. J Classif. 1985;2(1):193–218. doi: 10.1007/BF01908075

[82] Pomponio R, Erus G, Habes M, Doshi J, Srinivasan D, Mamourian E, et al. Harmonization of large MRI datasets for the analysis of brain imaging patterns throughout the lifespan. Neuroimage. 2020;208:116450. doi: 10.1016/j.neuroimage.2019.116450 31821869 PMC6980790

[83] Bayley N. Bayley scales of infant and toddler development. 3rd ed. San Antonio, TX: Harcourt Assessment; 2006.

[84] Cox JL, Holden JM, Sagovsky R. Detection of postnatal depression. Development of the 10-item Edinburgh Postnatal Depression Scale. Br J Psychiatry. 1987;150:782–6. doi: 10.1192/bjp.150.6.782 3651732

[85] Nichols TE, Holmes AP. Nonparametric permutation tests for functional neuroimaging: a primer with examples. Hum Brain Mapp. 2002;15(1):1–25. doi: 10.1002/hbm.1058 11747097 PMC6871862

[86] Newman MEJ. Fast algorithm for detecting community structure in networks. Phys Rev E Stat Nonlin Soft Matter Phys. 2004;69(6 Pt 2):066133. doi: 10.1103/PhysRevE.69.066133 15244693

[87] Suárez LE, Markello RD, Betzel RF, Misic B. Linking structure and function in macroscale brain networks. Trends Cogn Sci. 2020;24(4):302–15. doi: 10.1016/j.tics.2020.01.008 32160567

[88] Mišić B, Betzel RF, de Reus MA, van den Heuvel MP, Berman MG, McIntosh AR, et al. Network-level structure-function relationships in human neocortex. Cereb Cortex. 2016;26(7):3285–96. doi: 10.1093/cercor/bhw089 27102654 PMC4898678

[89] Hawrylycz MJ, Lein ES, Guillozet-Bongaarts AL, Shen EH, Ng L, Miller JA, et al. An anatomically comprehensive atlas of the adult human brain transcriptome. Nature. 2012;489(7416):391–9. doi: 10.1038/nature11405 22996553 PMC4243026

[90] Zamani Esfahlani F, Faskowitz J, Slack J, Mišić B, Betzel RF. Local structure-function relationships in human brain networks across the lifespan. Nat Commun. 2022;13(1):2053. doi: 10.1038/s41467-022-29770-y 35440659 PMC9018911

[91] van den Heuvel MP, Kersbergen KJ, de Reus MA, Keunen K, Kahn RS, Groenendaal F. The neonatal connectome during preterm brain development. Cereb Cortex. 2015;25(9):3000–13. doi: 10.1093/cercor/bhu095 24833018 PMC4537441

[92] Song L, Mishra V, Ouyang M, Peng Q, Slinger M, Liu S, et al. Human fetal brain connectome: structural network development from middle fetal stage to birth. Front Neurosci. 2017;11:561. doi: 10.3389/fnins.2017.00561 29081731 PMC5645529

[93] Chen R, Zhao R, Li H, Xu X, Li M, Zhao Z, et al. Development of the fetal brain corticocortical structural network during the second-to-third trimester based on diffusion MRI. J Neurosci. 2024;44(29):e1567232024. doi: 10.1523/JNEUROSCI.1567-23.2024 38844343 PMC11255424

[94] Yap P-T, Fan Y, Chen Y, Gilmore JH, Lin W, Shen D. Development trends of white matter connectivity in the first years of life. PLoS One. 2011;6(9):e24678. doi: 10.1371/journal.pone.0024678 21966364 PMC3179462

[95] Tymofiyeva O, Hess CP, Ziv E, Lee PN, Glass HC, Ferriero DM, et al. A DTI-based template-free cortical connectome study of brain maturation. PLoS One. 2013;8(5):e63310. doi: 10.1371/journal.pone.0063310 23675475 PMC3652871

[96] Wen X, Zhang H, Li G, Liu M, Yin W, Lin W, et al. First-year development of modules and hubs in infant brain functional networks. Neuroimage. 2019;185:222–35. doi: 10.1016/j.neuroimage.2018.10.019 30315911 PMC6289727

[97] Haber SN. Corticostriatal circuitry. Dialogues Clin Neurosci. 2016;18(1):7–21. doi: 10.31887/DCNS.2016.18.1/shaber 27069376 PMC4826773

[98] Alcauter S, Lin W, Smith JK, Short SJ, Goldman BD, Reznick JS, et al. Development of thalamocortical connectivity during infancy and its cognitive correlations. J Neurosci. 2014;34(27):9067–75. doi: 10.1523/JNEUROSCI.0796-14.2014 24990927 PMC4078084

[99] Buneo CA, Jarvis MR, Batista AP, Andersen RA. Direct visuomotor transformations for reaching. Nature. 2002;416(6881):632–6. doi: 10.1038/416632a 11948351

[100] Fenchel D, Dimitrova R, Robinson EC, Batalle D, Chew A, Falconer S, et al. Neonatal multi-modal cortical profiles predict 18-month developmental outcomes. Dev Cogn Neurosci. 2022;54:101103. doi: 10.1016/j.dcn.2022.101103 35364447 PMC8971851

[101] Donahue CJ, Sotiropoulos SN, Jbabdi S, Hernandez-Fernandez M, Behrens TE, Dyrby TB, et al. Using diffusion tractography to predict cortical connection strength and distance: a quantitative comparison with tracers in the monkey. J Neurosci. 2016;36(25):6758–70. doi: 10.1523/JNEUROSCI.0493-16.2016 27335406 PMC4916250

[102] Ercsey-Ravasz M, Markov NT, Lamy C, Van Essen DC, Knoblauch K, Toroczkai Z, et al. A predictive network model of cerebral cortical connectivity based on a distance rule. Neuron. 2013;80(1):184–97. doi: 10.1016/j.neuron.2013.07.036 24094111 PMC3954498

[103] Horvát S, Gămănuț R, Ercsey-Ravasz M, Magrou L, Gămănuț B, Van Essen DC, et al. Spatial embedding and wiring cost constrain the functional layout of the cortical network of rodents and primates. PLoS Biol. 2016;14(7):e1002512. doi: 10.1371/journal.pbio.1002512 27441598 PMC4956175

[104] Theodoni P, Majka P, Reser DH, Wójcik DK, Rosa MGP, Wang X-J. Structural attributes and principles of the neocortical connectome in the marmoset monkey. Cereb Cortex. 2021;32(1):15–28. doi: 10.1093/cercor/bhab191 34274966 PMC8634603

[105] Noori HR, Schöttler J, Ercsey-Ravasz M, Cosa-Linan A, Varga M, Toroczkai Z, et al. A multiscale cerebral neurochemical connectome of the rat brain. PLoS Biol. 2017;15(7):e2002612. doi: 10.1371/journal.pbio.2002612 28671956 PMC5507471

