# Supplementary material for: Brain structural and functional connectivity converge prenatally but diverge after birth
Source: PLoS Biol. 2026 Sep 9;24(9):e3003927. doi: 10.1371/journal.pbio.3003927 (PMC13557396; doi:10.1371/journal.pbio.3003927)
Supplement: Supplementary file 1 [file pbio.3003927.s001.docx]

S1 Methods

**Image Preprocessing.** For the rs-fMRI data in the BCP cohort, each frame was registered to the single-band reference (SBRef) image using a 6 DOF transformation to correct for subject head motion. If the SBRef image was unavailable, the first frame was used as the reference. EPI distortion was subsequently corrected using the TOPUP tool in FSL (https://fsl.fmrib.ox.ac.uk/fsl), based on a pair of scans with opposite phase-encoding directions. The functional image was then linearly registered to the corresponding T1-weighted image, followed by high-pass temporal filtering. Independent component analysis (ICA) was performed to decompose the BOLD signal into 150 components. A trained deep learning model was then applied to identify and remove noise-related components. Nuisance covariate regression was preformed to remove head motion, as well as white matter, CSF, and whole brain signals. Only rs-fMRI data that successfully passed the entire preprocessing pipeline and exhibiting no severe head motion were included in subsequent analysis.

The dMRI data of the BCP cohort were first denoised and corrected for Gibbs ringing artifacts using MRtrix3 (https://www.mrtrix.org/). EPI distortion was estimated using images acquired with opposite phase encoding directions via the TOPUP tool in FSL, and the resulting field map was subsequently applied in the EDDY command to jointly perform eddy current correction and slice-to-volume motion correction. B1 field inhomogeneity was then corrected across the dMRI volume series. The preprocessed dMRI data were linearly registered to T1 space using FILRT and upsampled to an isotropic resolution of 1 mm. Only dMRI data passing the complete preprocessing pipeline and successfully registered to T1 space were included in subsequent analysis.

**Cortical Parcellation Registration.** For the dHCP population, cortical parcellation was first mapped from the dHCP 40-week template surface [1] to individual surface using the provided transformation, and then projected to individual volume space via the Connectome Workbench (<https://www.humanconnectome.org/software/workbench-command>).

In the HCP population, cortical parcellation was first mapped from the dHCP 40-week template surface to the HCP S1200 template surface [2] using the transformation provided by a prior study [3], and subsequently to individual surface and volume space.

For the BCP dataset, a cortical surface of the BCP 0-month template [4] was first generated from tissue segmentation using the dHCP surface reconstruction pipeline [5]. Cortical parcellation [6] was then registered from the dHCP 40-week template surface [1] to the BCP 0-month template surface [4] using the Multimodal Surface Matching (MSM) toolbox [7], and manually refined by two experienced neuroradiologists (ZRK and LMY). The surface-based parcellation was subsequently projected into volumetric space using the Connectome Workbench. Next, the parcellation in the BCP 0-month volumetric template was nonlinearly registered to age-specific templates for other months via the rigid, affine, and symmetric diffeomorphic deformable (SyN) [8] registration implemented in ANTs (<https://github.com/ANTsX/ANTs>). All template parcellations were visually inspected and manually adjusted when necessary. Finally, for each participant, the age-matched template [4] was registered to individual space via SyN [8] registration.

**Network Metrics.** The weighted participation coefficient $P_{i}^{w}$ of node $i$ in a weighted network is defined as [9]

$$1-\sum_{m\in M} \left( \frac{k_{i}^{w}(m)}{k_{i}^{w}} \right)^{2}$$

where $M$ is the set of modules, predefined according to Yeo’s functional sub-networks, and $k_{i}(m)$ is the sum of positive (or negative) connections between node $i$ and all nodes within module $m$. The positive and negative participation coefficients of a node was then averaged.

The modularity $Q$ of a weighted network is defined as [10]

$$Q= \frac{1}{l^{w}}\sum_{i,j\in N} \left[ w_{ij}-\frac{k_{i}^{w}k_{j}^{w}}{l^{w}} \right]\delta_{m_{i},m_{j}}$$

where $l^{w}$ is the sum of all weights in the network, $w_{ij}$ is the connection weight between node $i$ and $j$, $k_{i}^{w}$ and $k_{j}^{w}$ are the weighted degree of node $i$ and $j$, $m_{i}$ is the module containing node $i$, and $\delta_{m_{i},m_{j}}=1$ if $m_{i}=m_{j}$, and 0 otherwise. In this study, negative weights are treated in the same way as positive weights in the signed functional networks. A Louvain-like locally greedy algorithm was used as a heuristic to maximize this modularity quality index subject to a partition $M$ of nodes into communities. For each network, the Louvain algorithm was run 100 times, and the average $Q$ corresponding to the most frequently occurring number of modules was taken as the final $Q$.

**Non-negative Matrix Factorization.** The definitions for metrics we used to determine the optimal number of components in NMF is list below:

1) Silhouette coefficient.

The silhouette coefficient measures how well each region fits within its assigned cluster compared to other clusters [11]. For region $i$, the silhouette coefficient $s(i)$ is defined as

$$s\left( i \right)=\frac{b\left( i \right)-a(i)}{max\left\{ a\left( i \right),b(i) \right\}}$$

where $a(i)$ is the average dissimilarity between region $i$ and all other regions within the same cluster (intra-cluster), and $b(i)$ is the minimum average dissimilarity between region $i$ and regions in any other cluster (inter-cluster). The silhouette coefficient ranges from -1 to 1, with values close to 1 indicating that a region is well-matched to its own cluster and poorly matched to other clusters, whereas values below 0 suggest that the region might be assigned to the wrong cluster. The whole brain silhouette coefficient is the average of all region values.

2) Adjusted rand index

ARI is a commonly used validation metric in clustering tasks to evaluate the similarity between two results (ground truth labels and prediction labels), adjusted for chance [12]. We used the mean Adjusted Rand Index (ARI) across 100 half-split bootstraps as the metric for evaluating reproducibility, and the standard deviation of ARI across bootstraps as metric for evaluating stability.

**Developmental Trajectory Modeling.** To examine age-related changes in SF coupling and network metrics, we employed a Generalized Additive Mixed Model (GAMM), which uses non-parametric estimation to capture both linear and nonlinear developmental trajectories.

In the dHCP dataset, the GAMM included gender, mean frame-wise displacement (FD, for SF coupling and FC network metrics), prematurity (born before 37 weeks or not), and subject ID as covariates. Continuous variables, including scan age and FD, were modeled as smooth terms using cubic spline basis functions, whereas categorical variables (gender and prematurity) were modeled as linear terms. Subject ID was included as a random intercept. For the BCP dataset, covariates were modeled in the same as in the dHCP dataset, except that prematurity was excluded, as all BCP participants were full-term born, and the dMRI protocol was additionally included as a covariate for SF coupling and SC network metrics. To estimate the growth rate, we applied the finite difference method to approximate the derivative of the age smooth term.

References

1.	Bozek J, Makropoulos A, Schuh A, Fitzgibbon S, Wright R, Glasser MF, et al. Construction of a neonatal cortical surface atlas using Multimodal Surface Matching in the Developing Human Connectome Project. Neuroimage. 2018;179:11-29. doi: <https://doi.org/10.1016/j.neuroimage.2018.06.018>.

2.	Van Essen DC, Smith SM, Barch DM, Behrens TEJ, Yacoub E, Ugurbil K. The WU-Minn Human Connectome Project: An overview. Neuroimage. 2013;80:62-79. doi: <https://doi.org/10.1016/j.neuroimage.2013.05.041>.

3.	Williams LZJ, Fitzgibbon SP, Bozek J, Winkler AM, Dimitrova R, Poppe T, et al. Structural and functional asymmetry of the neonatal cerebral cortex. Nat Hum Behav. 2023;7(6):942-55. doi: 10.1038/s41562-023-01542-8.

4.	Chen L, Wu Z, Hu D, Wang Y, Zhao F, Zhong T, et al. A 4D infant brain volumetric atlas based on the UNC/UMN baby connectome project (BCP) cohort. Neuroimage. 2022;253. doi: <https://doi.org/10.1016/j.neuroimage.2022.119097>.

5.	Makropoulos A, Robinson EC, Schuh A, Wright R, Fitzgibbon S, Bozek J, et al. The developing human connectome project: A minimal processing pipeline for neonatal cortical surface reconstruction. Neuroimage. 2018;173:88-112. doi: <https://doi.org/10.1016/j.neuroimage.2018.01.054>.

6.	Li M, Xu X, Cao Z, Chen R, Zhao R, Zhao Z, et al. Multi-modal multi-resolution atlas of the human neonatal cerebral cortex based on microstructural similarity. Neuroimage. 2023;272. doi: <https://doi.org/10.1016/j.neuroimage.2023.120071>.

7.	Robinson EC, Jbabdi S, Glasser MF, Andersson J, Burgess GC, Harms MP, et al. MSM: A new flexible framework for Multimodal Surface Matching. Neuroimage. 2014;100:414-26. doi: <https://doi.org/10.1016/j.neuroimage.2014.05.069>.

8.	Avants BB, Epstein CL, Grossman M, Gee JC. Symmetric diffeomorphic image registration with cross-correlation: Evaluating automated labeling of elderly and neurodegenerative brain. Med Image Anal. 2008;12(1):26-41. doi: <https://doi.org/10.1016/j.media.2007.06.004>.

9.	Guimerà R, Amaral LAN. Cartography of complex networks: modules and universal roles. J Stat Mech-Theory Exp. 2005;2005(02). doi: 10.1088/1742-5468/2005/02/P02001.

10.	Newman MEJ. Modularity and community structure in networks. Proc Natl Acad Sci U S A. 2006;103(23):8577-82. doi: 10.1073/pnas.0601602103.

11.	Rousseeuw PJ. Silhouettes: A graphical aid to the interpretation and validation of cluster analysis. J Comput Appl Math. 1987;20:53-65. doi: <https://doi.org/10.1016/0377-0427(87)90125-7>.

12.	Hubert L, Arabie P. Comparing partitions. J Classif. 1985;2(1):193-218. doi: 10.1007/BF01908075.
