# Supplementary material for: Brain structural and functional connectivity converge prenatally but diverge after birth
Source: PLoS Biol. 2026 Sep 9;24(9):e3003927. doi: 10.1371/journal.pbio.3003927 (PMC13557396; doi:10.1371/journal.pbio.3003927)
Supplement: Supplementary file 2 [file pbio.3003927.s021.docx]

S1 Table Association between SF coupling and developmental outcomes at 18 months

|  | Cluster | Beta (β) | 95% CI | P (Permutation) |
| --- | --- | --- | --- | --- |
| Cognitive | Sensorimotor | 0.187 | [0.061 0.313] | **0.007** |
|  | High-order | 0.005 | [-0.124 0.134] | 0.960 |
|  | Visual | 0.140 | [0.011 0.268] | 0.077 |
| Language | Sensorimotor | 0.160 | [0.033 0.287] | **0.032** |
|  | High-order | -0.021 | [-0.151 0.108] | 0.799 |
|  | Visual | 0.106 | [-0.024 0.235] | 0.201 |
| Motor | Sensorimotor | 0.063 | [-0.064 0.191] | 0.427 |
|  | High-order | -0.066 | [-0.194 0.062] | 0.431 |
|  | Visual | -0.006 | [-0.135 0.123] | 0.943 |
| Receptive language | Sensorimotor | 0.121 | [-0.006 0.248] | 0.106 |
|  | High-order | -0.028 | [-0.156 0.101] | 0.756 |
|  | Visual | 0.110 | [-0.018 0.239] | 0.176 |
| Expressive language | Sensorimotor | 0.173 | [0.045 0.300] | **0.020** |
|  | High-order | -0.009 | [-0.140 0.121] | 0.922 |
|  | Visual | 0.081 | [-0.049 0.211] | 0.338 |
| Fine Motor | Sensorimotor | 0.077 | [-0.050 0.204] | 0.318 |
|  | High-order | -0.034 | [-0.162 0.094] | 0.695 |
|  | Visual | 0.035 | [-0.093 0.164] | 0.687 |
| Gross Motor | Sensorimotor | 0.016 | [-0.114 0.145] | 0.861 |
|  | High-order | -0.078 | [-0.208 0.051] | 0.349 |
|  | Visual | -0.051 | [-0182 0.079] | 0.548 |
